# Predicting base editing outcomes using position-specific sequence determinants

**DOI:** 10.1093/nar/gkac161

**Published:** 2022-03-14

**Authors:** Ananth Pallaseni, Elin Madli Peets, Jonas Koeppel, Juliane Weller, Thomas Vanderstichele, Uyen Linh Ho, Luca Crepaldi, Jolanda van Leeuwen, Felicity Allen, Leopold Parts

**Affiliations:** Wellcome Sanger Institute, Hinxton, UK; Wellcome Sanger Institute, Hinxton, UK; Wellcome Sanger Institute, Hinxton, UK; Wellcome Sanger Institute, Hinxton, UK; Wellcome Sanger Institute, Hinxton, UK; Center for Integrative Genomics, University of Lausanne, Lausanne, Switzerland; Wellcome Sanger Institute, Hinxton, UK; Center for Integrative Genomics, University of Lausanne, Lausanne, Switzerland; Wellcome Sanger Institute, Hinxton, UK; Wellcome Sanger Institute, Hinxton, UK; Department of Computer Science, University of Tartu, Tartu, Estonia

## Abstract

CRISPR/Cas base editors promise nucleotide-level control over DNA sequences, but the determinants of their activity remain incompletely understood. We measured base editing frequencies in two human cell lines for two cytosine and two adenine base editors at ∼14 000 target sequences and find that base editing activity is sequence-biased, with largest effects from nucleotides flanking the target base. Whether a base is edited depends strongly on the combination of its position in the target and the preceding base, acting to widen or narrow the effective editing window. The impact of features on editing rate depends on the position, with sequence bias efficacy mainly influencing bases away from the center of the window. We use these observations to train a machine learning model to predict editing activity per position, with accuracy ranging from 0.49 to 0.72 between editors, and with better generalization across datasets than existing tools. We demonstrate the usefulness of our model by predicting the efficacy of disease mutation correcting guides, and find that most of them suffer from more unwanted editing than pure outcomes. This work unravels the position-specificity of base editing biases and allows more efficient planning of editing campaigns in experimental and therapeutic contexts.

## INTRODUCTION

The CRISPR/Cas toolkit has enabled increasingly fine control over DNA sequences (1). This technology has already uncovered myriad findings in basic research, identified new cancer targets, and offered novel therapeutic avenues for genetic disorders (2–5). However, the limitation of generating only insertions and deletions without templated repair, and the stochasticity of outcomes have motivated the development of alternative effector proteins such as base editors (6–10) for more precise genome manipulation.

Base editors reduce the range of mutations generated by Cas9 to primarily base substitutions, and alter DNA without potentially apoptosis-inducing double strand breaks (11,12). They consist of a catalytically impaired Cas9 fused to a deaminase and domains that modulate the DNA repair pathways (8,9). There are two main classes of base editor: adenine base editors that convert adenines into guanines using an adenosine deaminase, and cytosine base editors that convert cytosines into thymines using a cytidine deaminase (13). Once at the target determined by the gRNA, the base editor deaminates suitable nucleotides, which are then converted to another base via DNA repair. The original reports of feasibility (8,9) have been built on to develop increasingly precise and active enzymes (13–18), and also to expand to C to G editing (19,20).

While powerful, base editors have variable efficacy across loci and within the target (8,9,21,22), as well as unintended activity (17,21–26). The window of activity for the most popular base editors is in positions 4 to 8 of the target sequence (8,9,21,22) (‘canonical window’), where the protospacer-adjacent motif is at position 21–23. First reports have attributed some of the variability of base editing efficacy across loci to the APOBEC deaminating domain (27,28), which has a preferred TCW sequence motif, but other sources of variation are less understood. Unintended edits can be frequent, both via off-target editing at unintended locations in the genome (17,23–26), and via bystander editing of bases near the target (21,22,26,29). Both types of unintended edits depend on the targeted sequence and position within it (8,9,21,22,24,26). However, the interplay of position, sequence, and other features that drive variation in editing rate, and could help predict editing outcomes, remains poorly characterized.

Here, we measure the editing frequency of two cytosine base editors (BE4GamRA (30) and FNLS (30)) and two adenine base editors (ABE8e (31) and ABE20m (18)) at thousands of target sites, and uncover new sequence biases that strongly confound the known position-specific editing rates. We use this understanding to build position-specific models of base editing and present a new tool that accurately predicts editing frequency across a range of datasets.

## MATERIALS AND METHODS

### Cell culture and cell line generation

K562 cells were cultured in RPMI, HEK293FT cells were cultured in Advanced DMEM (Gibco), and HAP1 cells were cultured in IMDM with GlutaMAX (ThermoFisher, cat. no. 31980048). In all cases, supplemented with 10% FCS, 100 U/ml penicillin and 100 mg/ml streptomycin. K562 and HEK293FT media were further supplemented with 2 mM glutamine. Cells were cultured at 37°C, 5% CO_2_. K562 cells endogenously expressing BE4 and FNLS were generated by infecting K562 cells with a lentiviral vector carrying a base editor and puromycin resistance genes (pLenti-BE4GamRA-P2A-Puro, Addgene 112673; pLenti-FNLS-P2A-Puro, Addgene 110841) (30). Lentivirus was produced and wildtype K562 cells were infected as described below. Twenty-four hours later, selection with 2 μg/ml puromycin was started. After 1 week, the selection was stopped and cells were expanded. After 10 days of expansion, cells were treated for an additional 3 days with 0.5 μg/ml puromycin to enrich for cells with desired constructs.

### Lentiviral library

The lentiviral library used in this study was the same one used in Allen *et al.* (32). Briefly, the library uses the pKLV2-U6gRNA5-PGKpuro2ABFP-W (33) backbone and encodes 41 630 gRNA–target constructs. The gRNA is 20 nt, with the first base of the gRNA always being a G to improve expression from the U6 promoter (leaving a 19 nt of match with the target), and the complete target construct comprising the spacer, PAM, and additional sequence context is 79 nt, flanked with PCR priming sites.

### Lentivirus production and titration

Lentivirus was produced using HEK293FT cells that were transfected with Lipofectamine LTX (Invitrogen). 5.4 μg of a lentiviral vector, 5.4 μg of psPax2 (Addgene 12260), 1.2 μg of pMD2.G (Addgene 12259) were mixed in 3 ml Opti-MEM together with 12 μl PLUS reagent and incubated for 5 min at room temperature. 36 μl of the LTX reagent was added and the mix was incubated for another 30 min at room temperature. 3 ml of the transfection mix was then added to 80% confluent cells in 10 ml DMEM media in a 10-cm dish. After 48 h the supernatant was collected and stored at 4°C. Fresh media was added to the cells and harvested 24 h later. The supernatants from both harvests were mixed and centrifuged overnight at 6000g at 4°C and then for another 2 h at 20 000g. The supernatant was removed and the viral pellets were resuspended in DPBS resulting in 50× concentration of the virus. The virus was stored at −80°C. The procedure was scaled up accordingly for a larger production of virus.

For virus titration, K562 cells were seeded into a 96-well plate at 5 × 10^4^ cells/well. Increasing amounts of virus and 8 μg/ml polybrene (hexadimethrine bromide, Sigma) were added to each well. The plate was centrifuged at 1000g for 30 min at room temperature. The cells were resuspended and cultured for 72 h before harvesting for FACS analysis. The viral titer was estimated based on BFP+ cells and scaled up for the following screens. Data was analyzed by FlowJo.

### Screening integrated BE4 and FNLS lines

All cell lines were infected with the construct library aiming at a multiplicity of infection (MOI) of 0.8 and a coverage of 800×. Each cell line was infected twice and treated as two biological replicates. K562-BE4 and K652-FNLS cells were seeded at a concentration of 1.5 × 10^5^ cells/ml. Cells were cultured for 27 days and samples were harvested at 3, 6, 10, 14, 17, 21, 24 and 27 days post-infection. Cells were passaged to maintain higher coverage than at the time of infection. At 4 days post-infection, a subsample of cells was harvested for FACS analysis to estimate the MOI based on BFP + cells. The data was analysed with FlowJo.

### Screening ABE8e, ABE20m and BE4 with transient transfection

All cell lines were infected with the construct library aiming at a multiplicity of infection (MOI) of 0.8 and a coverage of 800×. Each cell line was infected twice and treated as two biological replicates. To screen, 293FT cells were cultured in media containing 2 μg/ml puromycin for one week to select for infected cells. 6 × 10^7^ cells were then seeded into six tissue culture dishes with 150 mm diameter in 20 ml media. Twenty-four hours later, the media was refreshed with 15 ml of media. Transfection mixes were prepared in two steps (protocol adapted from (34)). First, 16 ml of Opti-MEM was mixed with 72 μg of base editor encoding plasmid (BE4, Addgene #112673, ABE8e, Addgene #138489 or ABE8.20-m, Addgene #136300), 8 μg of pCS-GFP plasmid and 800 μl Plus reagent. Secondly, 16 ml Opti-MEM was mixed with 400 μl Lipofectamine 3000 (Invitrogen) and 1600 μl Lipofectamine LTX. The two solutions were mixed together, incubated for 30 min at room temperature and 3.2 ml of the transfection mix was transferred to each tissue culture plate. Forty-eight hours later, 15 ml of media was added to cells. After 14 h cells were harvested and a subsample of cells were used to check for transfection efficiency via flow cytometry. The data was analysed with FlowJo.

### Screening Target-AID

For the Target-AID experiments, gRNAs were cloned into pAT977-TargetAID (a gift from Charles Boone). This vector is based on pLentiCRISPRv2 (Addgene 52961), in which the Cas9 enzyme is replaced with the Target-AID base editor (35). Guide RNAs were cloned into the vector using a standard protocol (36) and obtained plasmids were verified by Sanger sequencing.

Lentivirus was produced using a slightly different procedure than the one described above. First, 4.2 × 10^6^ HEK293T cells were seeded in a 10-cm dish. The next day, 9 μg lentiviral vector (pAT977-TargetAID + gRNA), 9 μg psPax2 (Addgene 12260), and 0.9 μg pMD2.G (Addgene 12259) were added to 0.2 ml Opti-MEM and incubated for 5 min at room temperature. 54 μl TransIT-LT1 transfection reagent (Mirus Bio LLC) in 36 μl Opti-MEM was added and the transfection mixture was incubated for another 30 min at room temperature and subsequently added to the cells. The next morning, the medium was removed and replaced with DMEM containing 1% (w/v) bovine serum albumin. After another 24 h, the medium was collected, centrifuged for 4 min at 335g, and the supernatant containing the lentivirus was stored at −80°C.

To infect HAP1 cells, 1.5 × 10^6^ cells/well were seeded in a 12-well plate and polybrene (final concentration 8 μg/ml) and 1–1.5 ml lentivirus were added to the cells. The plate was centrifuged for 2 h at 640g and then incubated at 35°C (5% CO_2_) for 4–6 h. Next, cells were collected, divided over two wells, and incubated at 35°C (5% CO_2_). After 2 days, the medium was replaced with IMDM containing 1 μg/ml puromycin. Cells were expanded after another 2 days and collected 3 days later (7 days after infection). Cells were maintained at 35°C (5% CO_2_) during the experiment, as the Target-AID enzyme has higher activity at lower temperatures (35).

Genomic DNA was extracted from the collected cells using the QIAamp DNA Mini Kit according to the manufacturer's instructions. Target regions were amplified using primers in Supplementary Table S2 and Illumina sequencing adapters were added using PCR. Purified PCR products were sequenced on an Illumina MiSeq instrument using paired-end 150-nt reads. Nucleotide substitution frequencies were calculated using CRISPResso2 (37) (Supplementary Table S6).

### Sequencing library preparation

Genomic DNA extraction and sequencing library preparation for the main screens were done as described in Allen *et al.* (32). Briefly, to amplify the target sequence from the gDNA, primers P1 and P2 (Supplementary Table S1) were used with the Q5 Hot Start High-Fidelity 2X Master Mix (NEB). To ensure coverage for each sample, 416 μg of gDNA was used as template and each PCR reaction was run in 50 μl aliquots containing no more than 5 μg DNA. The PCR reaction was column-purified with the QIAquick PCR Purification Kit (Qiagen). Sequencing adaptors and barcodes were added with a second round of PCR using the KAPA HiFi HotStart ReadyMix (Roche), primers P3 and P4 (Supplementary Table S1) and 1 ng of template DNA. Amplicons were purified with Agencourt AMPure XP beads in 1.2:1 ratio (beads to PCR reaction volume), quantified with the Quant-iT™ High-Sensitivity dsDNA Assay Kit (Invitrogen). The amplicons for the BE4 and FNLS screens were sequenced using a NovaSeq S4 XP and the ABE8e and ABE20m screens using HiSeq 4000 (Supplementary Table S4 for accession numbers).

To measure mismatch rate between guides and targets, we amplified the target region together with the guide sequence from the genomic DNA in one of the cell pools above before editing occurred using primers P5 and P6 (Supplementary Table S1). The sequencing library was prepared as described before, using 104 μg of gDNA as template for the PCR. Sequencing adaptors and barcodes were added with a second round of PCR using the KAPA HiFi HotStart ReadyMix (Roche), primers P3 and P4 and 1 ng of template DNA. The libraries were sequenced with a HiSeq 2500 using paired end sequencing, such that the forward reads covered the guide region and the reverse reads covered the target.

### Data processing

We assigned reads to guides, and generated outcome profiles using the custom processing pipeline from Allen *et al.* (32). Outcome profiles for a guide are represented by pairs of mutations and the number of guide reads which had that unique mutation. For convenience, we also stored a matrix of the fraction of guide reads containing every possible base substitution at every position in the target sequence (12 substitutions × 79 positions). To ensure adequate coverage, we first removed guides with less than 100 reads in any sample (timepoint or replicate) from the analysis.

For BE4 and FNLS, we calculated the correlation of C to T editing at positions 4 to 8 between two replicate screens at each timepoint and found that replicates agreed with each other less at the later timepoints (Supplementary Figure S4B). We speculate that this is due to the toxicity of the editors, an argument that is supported by the decrease in average C to T editing at timepoints 21–27 (Supplementary Figure S4A). Thus, we chose to combine timepoints 10–17 (which were highly correlated, Supplementary Figure S4B) in our BE4 and FNLS data, by pooling together all the reads assigned to the same guide in each timepoint and treating this as a single screen. We then calculated the between-replicate correlation of C to T editing on the dataset of combined timepoints at positions 4 to 8, and found that replicates were very similar (median Pearson's *R* across positions of 0.87 and 0.91 in BE4 and FNLS, respectively, Supplementary Figure S1A and B).

For all editors, we combined the replicates using the same method as with timepoints, by adding read counts. We filtered for guides common to the relevant timepoints, removed guides with under 100 reads, and retained guides for which we had guide-target mismatch information, which left us with 14 409 guides.

To evaluate the consistency of screening using lentivirus and transient transfection to deliver the base editor, we calculated the correlation of C to T editing frequency using BE4 between the approaches. Given high reproducibility (Pearson's *R* of 0.87, Supplementary Figure S1E), we arbitrarily used results from BE4 delivered by lentivirus for downstream analyses.

### Correcting for mismatched guide–target pairs

To correct for recombination during infection of the guide library, which results in guide-target mismatch in some cells, we calculated the guide-target match rate for each guide using data from a long-range PCR on an early time point in one cell pool. The reads were assigned to guides by first checking if the forward read was a direct match to any of our guide sequences and, if matched, that read was assigned to that target. If the read was not a perfect match, we checked if the middle 10 bases of the read matched the middle 10 bases of any of our guides, as this stretch of bases could uniquely identify 82% of our guides. Once the forward PCR reads were assigned to guides, we aligned the expected target of the assigned guide with the sequence of the reverse read using the pairwise2 method from Biopython (38). Gaps were given a penalty of −1 and extending a gap was given a penalty of −0.1 (pairwise2.align.globalxs(guide1, guide2, −1, −0.1)). Random alignments of different targets in the dataset to the wrong guide all had scores below 41 (Supplementary Figure S4C), so we set a threshold of 50 to call a match, and any reads with an alignment score under this were considered mismatched from recombination. We calculated the guide-target match rate for a guide as the fraction of matched reads for that guide.

To correct for recombination-induced mismatch, we scaled the total number of reads in the base editing experiment for each guide by its guide-target match rate to get the number of reads that came from matching constructs. The number of reads with edits was left unchanged under the assumption that the constructs matched to be able to create an edit.

### Measures of gRNA efficacy

DeepSpCas9 scores and RuleSet2 scores were calculated using the 20 nucleotides of the target sequence. DeepCas9 scores were obtained from the batch prediction tool offered at http://deepcrispr.info/DeepSpCas9 with default settings, and RuleSet2 scores were computed using the software from (39), also with default settings. Empirically measured Cas9 mutation efficacy was obtained from (32), which used the same guide library as this study in K562 cells.

### Other base editing efficiency datasets

Data were downloaded from (22) and (21) and used to calculate editing rates for each substitution at each position for every guide. We used the mESC-BE4 and mESC-ABE datasets from (21) as the closest match to our editors, and because of their high concordance of replicates.

### Creating position-specific datasets

To make experiments comparable, we standardized real editing rates at each position by subtracting the mean edit rate at that position and dividing by the standard deviation at that position for each dataset. Guides were converted into feature vectors by first one-hot encoding the 20 nt guide sequence and then appending the melting temperature of the 20 nt guide sequence as calculated using the Biopython (38) MeltingTemp function.

### Train and test data split

We maintained the train and test dataset distinction provided in the Song *et al.* data, and randomly partitioned the guides both in our data and the Arbab *et al.* data into training and test sets with 90% for training and 10% for testing. Models were only trained on training sets and only evaluated on test sets.

To create a combined dataset, we appended the training sets of our BE4 dataset, our FNLS dataset, the mES-BE4 dataset from (21) and the cytosine dataset from (22) together to get a combined cytosine training dataset for each position. Similarly, we combined the training sets of our ABE8e dataset, our ABE20m dataset, the mES-ABE dataset from (21) and the adenine dataset from (22) to get a combined adenine training dataset for each position. The test sets for each editor type were also combined in this manner.

### Modelling editing rate

We trained and evaluated models with one-hot encoded sequence features and different sets of guide efficacy features on the training set, using 5-fold cross validation, to select the final set of features used. We found that all combinations of melting temperature, DeepSpCas9 score and RuleSet2 score produced similar contributions to predictive accuracy and functioned as proxies for each other, so we chose to use melting temperature alone.

We predicted standardized editing rate (raw rate linearly transformed to 0 mean and unit standard deviation) for base editing efficacy at each position using gradient-boosted trees, as implemented in the Scikit-learn package (40). One gradient-boosted tree model was trained per position. For each one, we chose to use 100 shallow trees (n_estimators), with maximum depth 4 (max_depth), a minimum of two samples per leaf node (min_samples_leaf) and a learning rate of 0.1. These values were obtained through 5-fold cross-validation on the training set, independently testing values more extreme than the selection in both directions (n_estimators 10–1000, max_depth 1–10, min_samples_leaf 1–50, learning rate 0.001–1). After training the final models on the full training set, we evaluated their performance on the test set by calculating the Pearson correlation between the predicted standardized rate and the measured standardized rate, as well as mean squared error.

### Model selection

We tested three kinds of models to predict editing rate: linear regression, gradient boosted trees, and neural networks. For the linear models, we used the LinearRegression function from the Scikit-learn package (40) and tested hyperparameter values of L1 and L2 regularization from 0.0001 to 1 using 5-fold cross-validation. Neural networks were implemented using the PyTorch package (41). We tested varying numbers of hidden layers from 1 to 5, and layers widths for each layer of 10 to 500. After training each in the manner described above, we observed that they had similar performances (Supplementary Figure S3A) and settled on the gradient boosted trees for their ability to combine features better than the linear models, as well as their increased interpretability relative to the neural networks.

### Downsampling experiments

In order to test whether our models were sensitive to the quantity of training data, we performed downsampling experiments. For each model, we sampled 100 new training sets for each of the fractions of 80%, 50%, 25% and 10% of the full training dataset randomly without replacement, and trained the model on these smaller sets. Performance was evaluated by calculating correlation between predicted and measured rates on the full test set.

### Comparisons with other models of editing rate

We compare FORECasT-BE to two other models, BE-HIVE (21) and DeepCBE (22). Predictions for DeepCBE were obtained using the batch prediction tool offered at http://deepcrispr.info/DeepBaseEditor/. Predictions for DeepABE were obtained by running the command-line tool downloaded from https://github.com/MyungjaeSong/Paired-Library/tree/DeepCRISPR.info/DeepBaseEditor. Predictions for BE-HIVE were obtained by running the BE-HIVE command-line tool from https://github.com/maxwshen/be_predict_efficiency. For BE-HIVE, we specified a mean of 0.5 and a standard deviation of 0.25 as the scaling parameters; these linearly scale the outputs and do not affect the correlations to true editing rates. When using the BE-HIVE model to evaluate guides from the Song *et al.* study, we padded the given 30nt sequences with As to create the required 50nt input.

### Endogenous data comparisons

Published endogenous base editing outcomes were obtained from Song *et al.* (22), Komor *et al.* (42) and Richter *et al.* (31). Novel data for Target-AID was generated as described above. Editing rates for each substitution at each position for every guide were calculated as described in ‘Creating position-specific datasets’, and predicted standardized edited rates as described above. To account for the different editing window of Target-AID, we shifted each Target-AID guide sequence forward by 3nt (such that the original position 1 became the new position 4) and then used our model on these shifted guides. Standardized predictions were transformed back into absolute efficiencies using per-position means and standard deviations. Means and standard deviations for the Song *et al.* data were obtained from the Song high-throughput screening dataset (22). For the remaining datasets, where reliable estimates could not be obtained with the same approach, we used a fixed mean and standard deviation for position 6 and then scaled this to other positions using the scaling observed in our data (Supplementary Table S3). The mean and standard deviation used for Komor *et al.* were 0.5 and 0.1, for Richter *et al.* 0.8 and 0.1 and for Target-AID 0.5 and 0.2.

When comparing purity of outcomes in the Song *et al.* endogenous data, we defined the guide's purity of editing at a given position as its fraction of reads with the intended edit at that position divided by the total fraction of its reads with any intended edit across all positions.

### Prediction in disease contexts

A set of guides targeting pathogenic SNPs correctable by a C to T or A to G substitution was obtained from (43). We predicted standardized efficiencies for positions 3–10 in this guide set and scaled them into real efficiencies by assuming a mean of 50% editing at position 6 to match the maximum rate in (21). We computed the predicted correction efficiency as the rate of C to T or A to G editing at the position of the SNP in the guide, and the expected number of unintended edits as the sum of predicted C to T or A to G editing at other positions.

## RESULTS

### Target base context and gRNA efficacy influence editing rate

**Figure 1.**
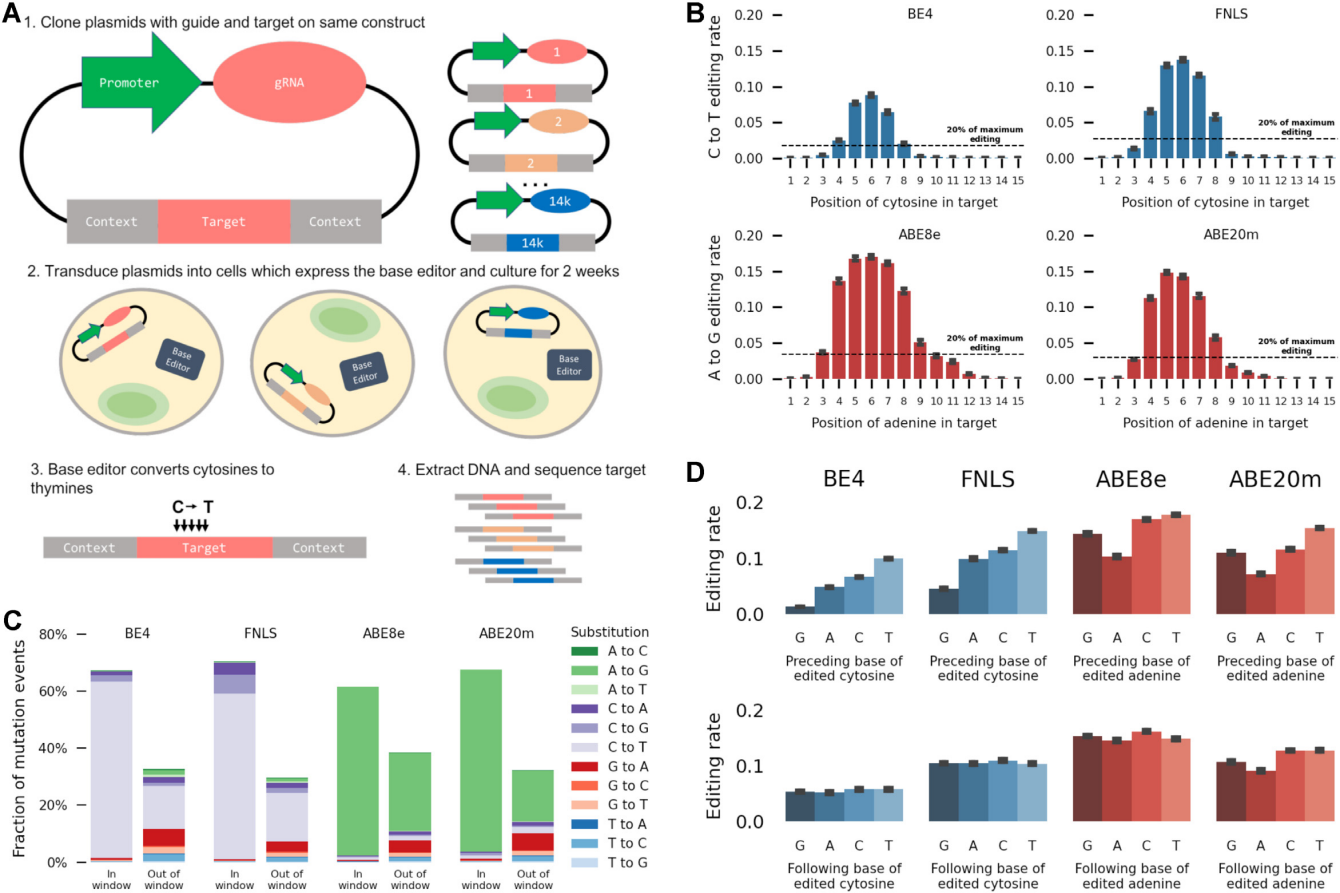
(**A**) A method for high throughput measurement of base editor outcomes. (1) Constructs containing both a gRNA and its target sequence (matched colors) in variable context (gray boxes) are cloned into target vectors containing a human U6 promoter (green). (2) The constructs are packaged into lentiviral particles and used to infect cells that either express base editor protein or have been transfected with base editor protein. (3) The base editors (C to T as an example here) generate base substitutions in the target. (4) DNA from cells is extracted, the target sequence and context are amplified with common primers, and the mutations in the target are determined by sequencing. (**B**) C to T and A to G editing is highest at positions 4–8 in the target sequence. Median C to T (blue bars) or A to G (red bars) editing activity (y-axis) at positions 1–15 in the target sequence (x-axis). Error bars: 95% confidence intervals from 1000 bootstrap samples. Black dashed line: 20% of the maximum editing value found at any position. (**C**) Intended edits in the canonical window are the most frequent outcome for both cytosine and adenine editors. Cumulative frequency (y-axis) of substitution type (color) in the canonical window (position 4–8, left bars) and outside (right bars) for all four editors (x-axis). (**D**) Median base editing rate in the canonical window across targets (y-axis) is influenced by preceding (top) or following (bottom) base (x-axis). Error bars: 95% confidence intervals from 1000 bootstrap samples.

We set out to quantify the sequence- and gRNA- dependent frequency of editing by cytosine and adenine base editors. We chose two cytosine base editors to screen: FNLS (30), a version of the BE3 editor with an altered nuclear localization signal, and BE4GamRA (30) (hereafter referred to as BE4), an optimization of BE4Gam (42). We also chose two adenine editors: ABE8e (31) and ABE8.20-m (18) (hereafter referred to as ABE20m), both directed evolutions of ABE7.10 (9) with mutations selected for increased editing efficiency. Following (32), we employed a library of self-targeting constructs which encode both a 23nt protospacer adjacent motif-endowed target sequence embedded within 56nt of randomized sequence context, and an expression cassette for a gRNA matching the target. After introducing these constructs into cells, and allowing editing to occur, we sequenced the targets (Figure 1A). We measured base editing frequency in the K562 and HEK293T human cell lines, with a median screen coverage of 890× for cytosine editors and 470× for adenine editors, and sequencing coverage of ∼1500× (Methods, Supplementary Data). After filtering, we recovered the fraction of edited reads (‘editing rate’) for each base of 14 409 target sequences, and observed excellent reproducibility between replicates (combined Pearson's R across all positions from 0.73 to 0.91, Supplementary Figure S1A–D).

We uncovered both known and novel biases in base editing outcomes. The median editing rate across targets was highest at position 6 of the sequence for all editors, and decreased with distance from this position (Figure 1B). Cytosines and adenines in the canonical window (positions 4 to 8) were substantially edited, with rates above 20% of that at position 6, while those outside the canonical window had rates below 10% (Figure 1B). The editing rate per cytosine did not change depending on the number of cytosines in the window for cytosine editors (editing rate per cytosine between 0.04 and 0.05 for BE4, Supplementary Figure S1F), but decreased with more editable adenines in the window for adenine editors (from 0.13 with a single adenine to 0.08 with six adenines in ABE8e, Supplementary Figure S1F). When multiple editable bases were present, editing rates were highly correlated for neighbouring bases (average Pearson's *R* = 0.74 across all editors, Supplementary Figure S1G), but only moderately correlated otherwise (average Pearson's *R* = 0.42 across all editors, Supplementary Figure S1G).

Unintended edits accounted for 35%–42% of all mutation events across editors (Figure 1C). The most frequent unintended editing event in cytosine editors were C to T edits outside the canonical window (15% and 17% of all mutation events in BE4 and FNLS, respectively). G to A editing outside the window was the second most common unintended edit in BE4 (6%) and was relatively frequent in FNLS as well (3.6%), consistent with editing on the opposite strand (Discussion). We also observed frequent transversion edits in cytosine editors, accounting for 6.8% of all mutation events in BE4 (3.5% C to A, 3.3% C to G) and 14.5% in FNLS (6.2% C to A, 8.3% C to G). The adenine editors had different biases, with the most common unintended edits in both being A to G and G to A outside the canonical window (27% and 4% in ABE8e, 17% and 5% in Abe20m). Transversion edits were less common in adenine editors, accounting for less than 1% of mutation events in both ABE8e and ABE20m. All remaining substitutions combined comprised less than 9% of mutation events in any editor, with less than 2% of the total each (Figure 1C), and their rates outside the window were consistent with background rates as measured in control cells without base editors (Supplementary Figure S1H). Finally, insertion and deletion frequency in the target window remained below 0.5% (Supplementary Figure S1I), and we do not consider them further. Overall, nearly two thirds of observed edits were the intended transitions in the canonical window, and the bias towards intended transitions was smaller outside of the window.

Besides position in the targeted sequence, the other known influences of editing frequency are the identity of flanking bases and the gRNA efficacy (8,21,22). For all editors, the rate of the intended substitution was highest when it was preceded by a thymine (155%, 70%, 25% and 52% increase compared to the other three bases, in BE4, FNLS, ABE8e and ABE20m, respectively; *t* test *P* < 10^−20^ in each), consistent with the editing motifs of APOBEC (28) and tadA (44). C to T editing by cytosine editors was lower when preceded by a guanine (82% and 63% decrease in BE4 and FNLS respectively, t test *P* < 10^−20^, Figure 1D). A to G editing by adenine editors was lowest when preceded by an adenine (37% and 44% decrease in ABE8e and ABE20m, *t* test *P* < 10^−4^, Figure 1D). Editing by all effectors increased when a cytosine followed the edited base and decreased when followed by an adenine. In general, the TNC motif was consistently amongst the best edited sequences.

Sequence identity was important for several unintended substitutions as well (Supplementary Figure S1J). In particular, C to G transversions by cytosine editors increased over 4-fold at the TCT motif, and were more frequent when the C was followed by a T. This effect was recently used to develop C to G editors elsewhere (45–47). Furthermore, there is evidence that the cytosine base editors also operate on the opposite strand, as G to A edits were found in much greater quantity in cytosine editors than in controls with only wild-type Cas9 (Figure 1C), and these edits also mirrored motif preferences, albeit with lesser effect on the opposite strand (300% increase in TC to TT in BE4, 50% increase in GA to AA). In summary, the base preceding the edited one has the strongest effect on editing activity for intended transitions, and the following base has an effect on transversions as well.

Measures of Cas9 gRNA efficacy were also informative about base editing efficacy. Predictions from two computational models of gRNA quality (DeepSpCas9 (48) and RuleSet2 (39)), as well as the empirically measured wtCas9 mutation efficiency (32) were correlated with C to T editing frequency in cytosine editors (Pearson's *R* = 0.13, 0.05 and 0.14, respectively in BE4; 0.11, 0.03, 0.12 for FNLS; *P* < 0.01 in both) and A to G frequency in adenine editors (Pearson's *R* = 0.10, 0.11 and 0.09 for ABE8e; 0.11, 0.11 and 0.12 in ABE20m; *P* < 0.01 in both). The top decile of guides as scored by DeepSpCas9 were edited 70% more frequently than the bottom decile in BE4 (35%, 11% and 20% in FNLS, ABE8e and ABE20m, respectively; *P* < 10^−20^ in all editors; Supplementary Figure S1L). Similarly, the RuleSet2 scores and measured Cas9 mutation efficacies were 27% and 62% higher respectively in the top decile of scores compared to the bottom one in BE4 (13% and 25% in FNLS; 23% and 9% in ABE8; 33% and 24% in ABE20m; *P* < 10^−20^ in all editors; Supplementary Figure S1L). Finally, for better expression from the U6 promoter, the first nucleotide of each guide RNA was changed to a guanine, as has been recommended for genome-wide screens (49). Targets with a G at position 1 therefore have an improved guide-target match, and this increased editing by 20% over targets that did not start with a G. Thus, we successfully captured independent gRNA- and sequence-dependent biases that affected editing rate, with magnitudes of known effects consistent with existing studies (21,22) (Supplementary Figure S2A–C).

### Sequence effect depends on edited position

**Figure 2.**
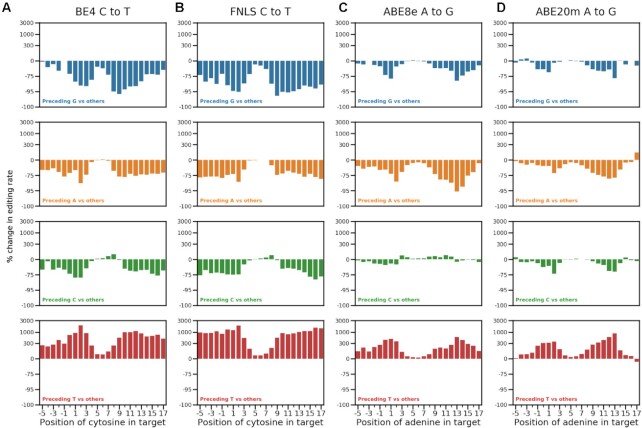
Position-dependent effect of the preceding base. Percentage change in editing rate (y-axis, logarithmic intervals) from having a base preceding the target base (color, rows) compared to all other bases, at different positions in the target sequence (x-axis) for all assayed editors (**A–D**).

**Figure 3.**
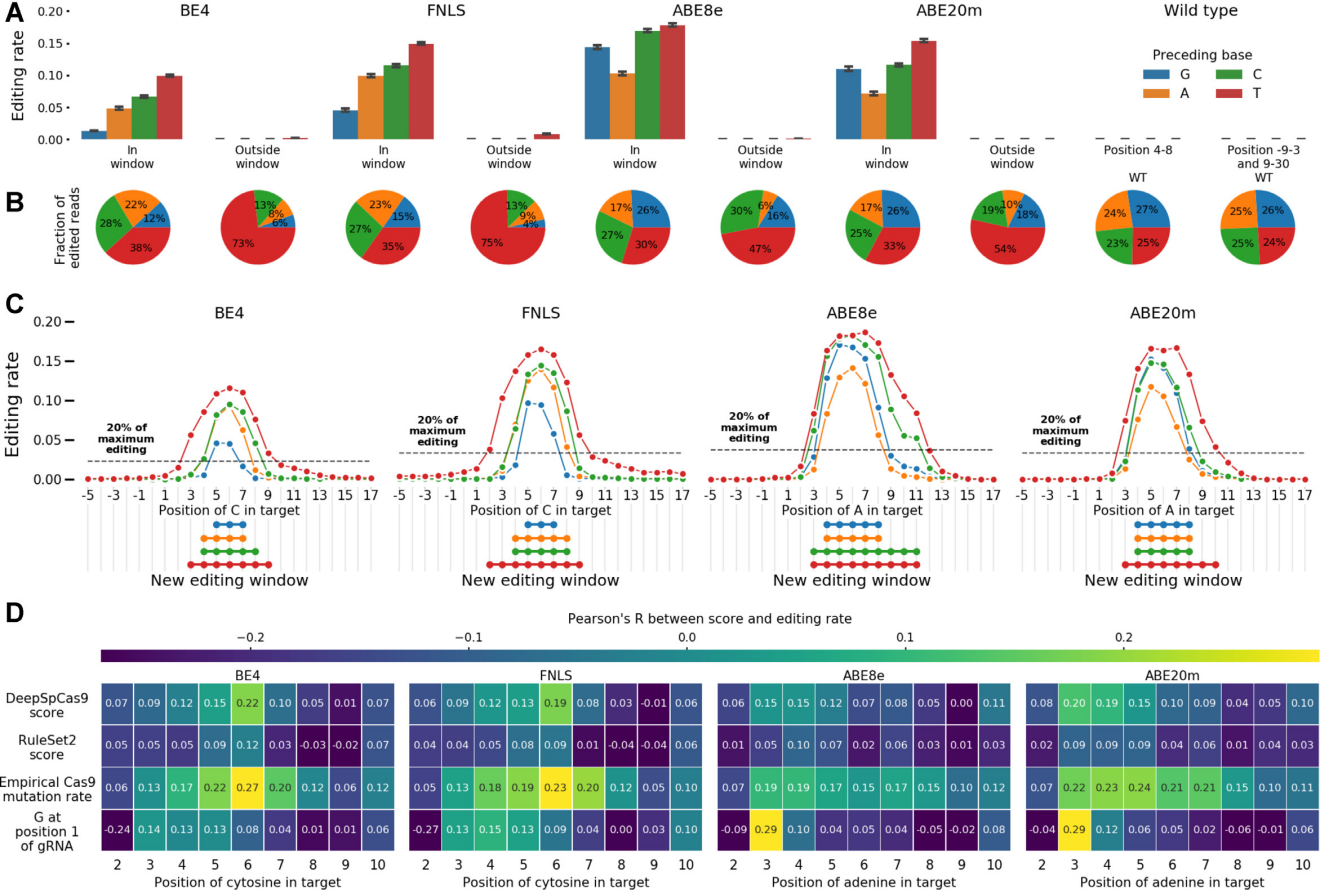
(**A**) Preceding thymines are a driver of out-of-window cytosine editing. Median editing rate across targets (y-axis), for every preceding base (colors), inside and outside canonical target window (x-axis), for cells with base editors (BE4, FNLS, ABE8e, ABE20m) and wild type ones without (WT) compared to the total fraction of C to T or A to G edited reads in the experiment. Canonical window: positions 4–8. Error bars: 95% confidence intervals from 1000 bootstrap samples. (**B**) Same as (a), but fraction of editing outcomes of the total. (**C**) Window of editing changes depending on the base preceding the edited one. Median editing rate (y-axis) of bases at positions −5 to 17 in the target (x-axis) for each preceding base type (colors) for each editor (panels). Black dashed line: 20% of the maximum editing rate at any position for all preceding bases. Linked dots: positions at which editing is above 20% of the maximum. (**D**) Correlation between gRNA quality and editing rate depends on the position. Pearson's R (color) between measures of gRNA quality (y-axis) and the position of the edited base in the target sequence (x-axis) for each editor (panels).

Surprisingly to us, the influence of flanking sequence on editing rate differed substantially across edited positions. For C to T edits using cytosine editors, the preceding guanine and thymine had the strongest marginal effect on editing rate (Figures 2 and 3A). The detrimental impact of a preceding G was larger away from the canonical window center, with a 49% decrease in median editing at position 6 in BE4, but an 89% decrease at position 9 compared to other preceding bases (35% and 93%, respectively, in FNLS; Figure 2). Similarly, a preceding T resulted in a 45% higher median editing rate at position 6, but a 1900% increase at position 2 (32% and 1800% in FNLS; Figure 2). Preceding thymines had a similar effect on A to G editing for adenine editors, with a 10% increase in editing at position 6 for ABE8e (22% for ABE20m) and 377% increase at position 2 (366% for ABE20m; Figure 2). Cumulatively, 38% of all C to T editing by BE4 across positions 4 to 8 was of the cytosine in the TC dinucleotide, but this increased to 73% for positions not in the 4 to 8 range, where activity was otherwise low (35% and 75% in FNLS; Figure 3B). This strong preference indicates the preceding thymine as a major driver of out-of-window cytosine editing. The preceding T effect was less prominent for the adenine editors, shifting the A to G editing rate from 30% in the window to 47% outside of it in ABE8e (33% and 54% in ABE20m, Figure 3B).

Position-dependent sequence biases were also present for unintended edits. C to G editing rate in cytosine editors was higher when preceded by a T (88% increase at position 5 and 1186% at position 9 for BE4, Supplementary Figure S2D, 71% and 1347% for FNLS, Supplementary Figure S2E), and lower with a preceding G (38% decrease at position 5, 93% decrease at position 9 for BE4, Supplementary Figure S2D, 28% and 96% for FNLS, Supplementary Figure S2E). In addition to the preceding base, a following T increased C to G editing across all positions in cytosine editors (173% increase at position 5 and 94% increase at position 9 in BE4, Supplementary Figure S2D). G to A editing is also increased by a following A, consistent with a TC motif on the opposite strand. Altogether, several unintended edits, especially in cytosine editors, exhibited substantial sequence bias that varied across the target (Supplementary Figure S2D–G).

The large variation in editing rate due to the preceding base suggests a more nuanced redefinition of the cytosine and adenine editing windows. A threshold of 20% of maximum editing for the target produces the canonical window for marginal editing, but gives different editing windows when stratifying by the preceding base. In cytosine editors, the window for cytosines preceded by Cs is positions 4–8, consistent with the canonical window, while a preceding A leads to a window of 4–7. However, with a preceding T, the window broadens to positions 3–9, and a preceding G shrinks it to positions 5–7 (Figure 3C). Similar trends hold for adenine editors, where the A to G window stretched from positions 3 to 11 when adenines were preceded by a T, but was reduced to positions 4 to 8 when preceded by an A or G (Figure 3C). These biases were also present in other large scale measurements of editing rates (21,22) (Supplementary Figure S2C).

Motivated by the position-dependent sequence effects, we next queried whether the impact of features that capture aspects of gRNA sequence and secondary structure also varies along the target. For cytosine editors, correlation between gRNA features and editing efficacy was strongest at position 6 (Pearson's R = 0.22, 0.12, 0.27 for DeepSpCas9 score, RuleSet2 score and measured Cas9 mutation efficacy respectively in BE4, Figure 3D), but declined with increasing distance from this position. Interestingly, this trend did not hold for the adenine editors, with the largest correlation between A to G editing and the metrics occurring earlier in the sequence for DeepSpCas9 and RuleSet2 scores, or staying consistent across the sequence for the measured Cas9 mutation efficacy (Figure 3D). These effects were also present in other datasets (Supplementary Figure S2C). Finally, a G at position 1 in the target was associated with increased editing at positions 3 and 4 in all editors and extending to positions 5 and 6 in cytosine editors (Figure 3D). These patterns of feature relevance suggest that gRNA features add bias to the already high editing rates at central positions (especially for cytosine editors), but are less relevant elsewhere. Conversely, sequence bias is lowest centrally, but dominates at outside positions.

### Accurate prediction of per-position editing

**Figure 4.**
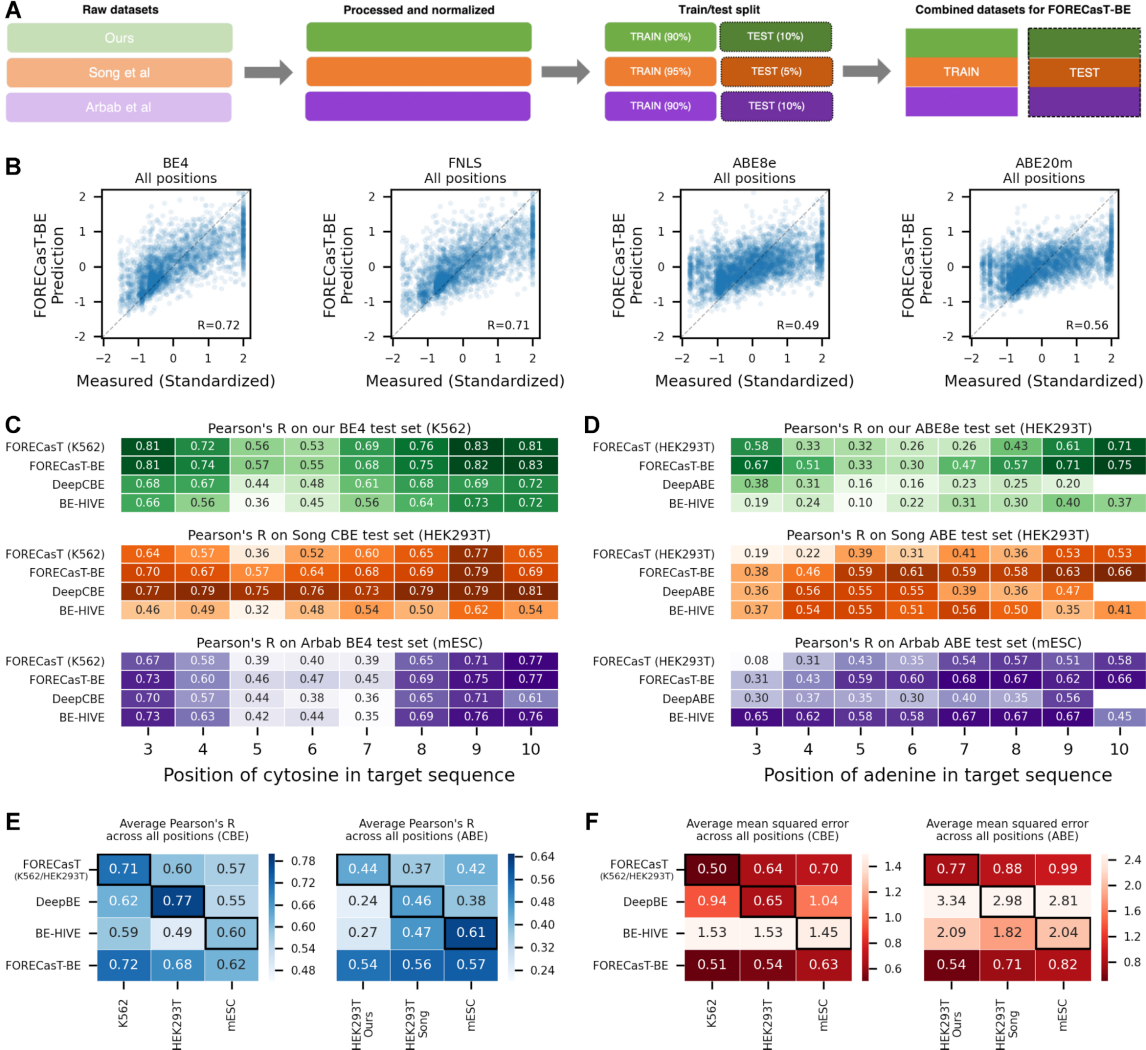
(**A**) Schematic of datasets used to train FORECasT-BE. Raw base editing efficiencies from this study (green), Song *et al.* (orange) and Arbab *et al.* (purple) are normalized independently to z-scores (opaque boxes), split into training and test sets (light and dark boxes), and combined into final training set and test sets employed by FORECasT-BE. (**B**) FORECasT-BE accurately predicts editing rate. Measured (x-axis) and predicted (y-axis) standardized editing rate (Methods) for guide RNAs (markers) in each editor we screened (panels). Dashed line: *y* = *x*. Label: Pearson's R between measured and predicted scores. (**C, D**) FORECasT-BE accurately predicts in a variety of contexts. Pearson's *R* between measurements and predictions of editing rates at different positions in the target (x-axis) for models (y-axis) trained on different cell types (panels) when using cytosine editing (C) or adenine editing (D) datasets. Models: FORECasT trained on only K562 (C) or HEK293T cells (D; top row), FORECasT-BE trained on combined dataset (second row), DeepCBE/DeepABE trained on HEK293T cells (third row) and BE-HIVE trained on mES cells (bottom row). Colors and datasets: as in (A). (**E, F**) FORECasT-BE generalizes well to many datasets. Average performance of models at all positions (y-axis) as measured using Pearson's *R* (E, blue heatmaps) and mean squared error (F, red heatmaps) when evaluated on a test set from one cell type (x-axis). Models: as in (C) and (D). Bold outlines: model and dataset from same publication. DeepBE: DeepABE or DeepCBE, depending on the dataset.

Given the improved understanding of position-dependent editing rates, we proceeded to build a position-specific editing model of base editing activity. We first split our data into training and test sets for each position (Figure 4A). Then, to better generalize across cell types, we combined our training data with that from previously published datasets (21,22), and trained FORECasT-BE, a gradient boosted tree model (50) (Supplementary Figure S3B), to predict the normalized editing frequency at positions 3–10 in the target sequence, as well as total fraction of reads edited at any position. Inputs to this model are nucleotide identities at each position in the guide and the melting temperature between the guide and target (Methods). When evaluated on the test set of guides from our experiment only, FORECasT-BE achieved a Pearson's *R* of 0.72 across all positions in BE4 (0.71, 0.49, 0.56 in FNLS, ABE8e and ABE20m, respectively, Figure 4B), with highest accuracies at outside positions (Supplementary Figure S3C). Feature importances in the model reflected the identified sequence biases, with the identity of the base preceding the edited one being most important (Supplementary Figure S3D–E). To determine whether the quantity of training data was a bottleneck to performance, we performed downsampling experiments where the training dataset was randomly reduced to 80%, 50%, 25% and 10% of the original size (Methods) and observe that performance plateaus at 80% of the full training set (Supplementary Figure S3F), suggesting that more training data would offer very marginal improvement. We incorporated FORECasT-BE into a command line tool (available at https://github.com/ananth-pallaseni/FORECasT-BE) and a web application (available at https://partslab.sanger.ac.uk/FORECasT-BE), which can be used to predict base editing rates for cytosine and adenine editors.

Recently, other methods have been developed for base editing rate prediction using cytosine and adenine editors: BE-HIVE (21), an autoregressive neural network, and DeepCBE/DeepABE (22), a convolutional neural network. Like FORECasT-BE, both models use the one-hot encoded guide sequence as input. We compared the performance of FORECasT-BE, which is a gradient-boosted tree, to these two models on each model's test set. We chose to use the BE-HIVE models for the BE4 and ABE editors, trained on measurements in mouse embryonic stem cells (mESCs; hereafter called the Arbab dataset), because the editors closest matched our own, and their mESC dataset was the most replicable of those reported. The DeepCBE/DeepABE models were trained on data from HEK293T cells (hereafter called the Song dataset). FORECasT-BE was trained on the training sets of both of these datasets as well as our own measurements in K562 and HEK293T cells. Each model predicts both a total editing rate per guide, as well as a set of specific outcomes, so we evaluate them separately for these tasks. First, we predicted the total fraction of reads containing any mutation across all positions, and observed improved performance of FORECasT-BE relative to other models (Pearson's R of 0.59 for cytosine editors and 0.59 for adenine editors for FORECasT-BE, 0.55 and 0.58 for BE-HIVE, and 0.54 and 0.13 for DeepCBE/ABE).

Next, we compared prediction performance of models trained on different datasets. To delineate the impact of model choice from the value of a broader training dataset, we also include a gradient boosted tree model like FORECasT-BE, but trained exclusively on our data (referred to hereafter as FORECasT-HEK293 or FORECasT-K562). Models trained on a single dataset perform better on that test set than models trained on different data (Figure 4C and D). For example, predictions of DeepCBE are best correlated to the measurements from Song HEK293T cytosine test dataset from its publication (Pearson's *R* = 0.76 at position 6 versus 0.64 for FORECasT-BE, the second-best model), but worst correlated to the measurements from the Arbab mESC BE4 dataset (Pearson's *R* = 0.38 at position 6, Figure 4C). FORECasT-BE, trained on multiple datasets, achieved at least 88% of the best Pearson's *R* for every dataset and position, and when averaged across positions, performed achieved at least 97% on cytosine editors and 112% on adenine editors (Figure 4E, Supplementary Figure S3G and H). Other models failed to generalize as well beyond their training dataset (average of 93% of best Pearson's R for DeepCBE, 72% for DeepABE, 82% for BE-HIVE CBE, and 88% for BE-HIVE ABE; Figure 4E, Supplementary Figure S3G and H). Finally, we compared each model using the mean squared error, which highlights the absolute differences between measured and predicted editing instead of the trend. Using this metric, FORECasT-BE often outperformed the other models even on the datasets on which they were built (Figure 4F, Supplementary Figure S3I and J). For example, the average mean squared error across all positions was 0.54 for FORECasT-BE on Song HEK293T cytosine data, but 0.65 for DeepCBE. Overall, we find that FORECasT-BE can accurately predict cytosine and adenine editing at par or better than existing models in a variety of high-throughput cell contexts.

**Figure 5.**
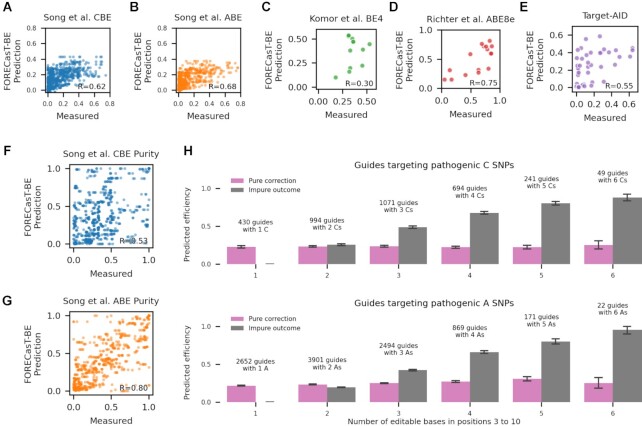
(**A–E**) FORECasT-BE accurately predicts position-specific editing rate in endogenous contexts. Measured (x-axis) and predicted (y-axis) editing rate (Materials and Methods) for guide RNAs (markers) at the different genomic positions tested in the Song *et al.* CBE dataset (A), Song *et al.* ABE dataset (B), Komor *et al.* BE4 dataset (C), Richter *et al.* ABE8e dataset (D), and a TARGET-AID dataset presented here (E, Materials and Methods). Label: Pearson's *R* between measured and predicted editing. (**F**, **G**) FORECasT-BE accurately predicts the purity of edited products in endogenous contexts. Measured (x-axis) and predicted (y-axis) purity of edits (Materials and Methods) across all editable positions (markers) for guide RNAs in the Song *et al.* CBE dataset (F) and Song *et al.* ABE dataset (G). Label: Pearson's R between measured and predicted purity. (**H**) Substantial editing of non-targeted cytosines and adenines for pathogenic corrections. Predicted fraction of impure edits in positions 3–10, (y-axis; grey bars) and edits that will correct the pathogenic SNP (y-axis; pink bars) for increasing number of editable cytosines (left) or adenines (right) in the window (x-axis). Error bars: 95% confidence intervals from 1000 bootstrap samples.

We next tested whether our model generalizes to measurements at endogenous target sequences. To do so, we gathered rates from experimental contexts presented in Song *et al.* (22) (170–230 guides per cell type, using CBEs and ABEs, in HCT116, HEK293T and U20S cells), Komor *et al.* (42) (six sites, using BE4, in HEK293T cells), Richter *et al.* (31) (7 sites, using ABE8e, in HEK293T cells), and a novel set of 15 sites screened using the TARGET-AID editor (35) in HAP1 cells (Methods). Pearson's *R* between predicted and observed editing in these datasets ranged from 0.30 to 0.75 (Figure 5A–E), similar to performance on the synthetic construct data used to train the model, and suggesting that the predictions generalize to edits in endogenous contexts.

An important use case for base editors is the correction of pathogenic SNPs in clinical contexts, which require pure edits for safety and efficacy. We first tested the ability of FORECasT-BE to predict the purity of an edit at a position (Materials and Methods) in endogenous data from (22) and achieved high accuracy with both cytosine and adenine editors (Pearson's *R* of 0.68 in cytosine editors, Figure 5F, 0.80 in adenine editors, Figure 5G). We then combined the FORECasT-BE predictions of purity and efficacy to evaluate potential of applying base editors to correct disease-relevant SNPs. To do so, we predicted base editing outcomes at 13,591 pathogenic SNPs from ClinVar which fall within positions 3–10 of a possible target, and can be converted with cytosine or adenine editors (43). We assumed a 50% conversion rate at position 6, which is the current expectation in a clinical scenario (21), and does not impact the resulting purities. Altogether, 64% of cytosine-targeting guides and 41% of adenine-targeting ones were predicted to have more combined editing of nearby cytosines or adenines than the targeted SNP (Figure 5H). The correlation between the expected number of unintended edits and the number of targetable bases in the window was 0.75 for cytosine-targeting guides and 0.76 for adenine-targeting guides, indicating that more targetable bases are expected to produce more unintended edits. While SNP correction purity is influenced by window position and nearby targetable bases (Supplementary Figure S3K), we identified 108 cytosine-targeting guides and 421 adenine-targeting guides that were predicted to correct the disease-relevant mutations with over 80% purity, and also were in the top 10% of correction efficiencies, and therefore may make reasonable therapeutic targets (Supplementary Table S5). For sites where this is not the case, our model can help predict the most frequent unintended edits, which can then be checked for their effect on the protein sequence.

## DISCUSSION

We reported strong position-dependent biases in the determinants of base editing rates from a large survey of editing outcomes in novel and published datasets of cytosine and adenine base editors. Our findings indicate a nuanced action of cytosine and adenine editing. The editing window depends on the preceding base, with TC or TA dinucleotides edited beyond the canonical positions 4 to 8. Transversion edits were moderately frequent, and also position-dependent with a strong bias from the following base. In general, as much as 30% of overall editing on average is either out-of-window or not the intended substitution, with out-of-window edits more frequent if following thymines. All the findings on position dependence also held in existing large-scale datasets that were generated using different base editor proteins, cell lines, and target libraries.

A key finding was the position-specificity of sequence feature influence. This is likely explained by the nature of the deaminating domain and the linker which connects it to the dCas9 in the base editor complex. The length of the linker determines which bases in the target the deaminase is proximal to, which creates the editing window. The central bases of the window are best positioned for deamination, while those further out require flexibility from the linker, resulting in lower effective local concentration, and thus less editing. The observed sequence preferences are then naturally determined by the preferred motifs of the deaminating domains. The structure of the APOBEC domain in cytosine editors, as well as the TadA domain in adenine editors, contains a pocket that best fits the TN dinucleotide. This motif was the strongest determinant of high editing rates, especially at outside positions where limited access to the deaminase means that editing might otherwise not occur at all. Conversely, central positions are edited more regardless of sequence motifs, so that gRNA efficacy and guide-target binding can become limiting, and their variation therefore has a greater influence on the observed rate.

We incorporated these insights into a predictive model, FORECasT-BE, and found it to be the most accurate for edits in our K562 and HEK293T data. In comparisons with published approaches, each model performed best on the dataset from its publication and did worse on the others, but FORECasT-BE performed very close to best in all tests. This quality is likely due to the diverse data used in its training, rather than any developments in model architecture or featurization. The same model trained on a single dataset performed as well as the model published for that data, including the drop in performance on other datasets. Additionally, while the input features to each model were nearly identical (one-hot encoded guide sequence and a proxy for gRNA–target duplex melting temperature), the model families differed substantially, with FORECasT-BE using gradient boosted trees and the other two using different neural networks. Since all these model types, as well as additional approaches we tested, performed similarly, it is likely that they make use of the same signal present in the measurements. We therefore speculate that further advances in base editing efficacy modelling will require assaying outcomes in a wider complement of contexts, systematically varying cell type, repair activity, effector protein, delivery mechanism, guide RNA expression method, etc. Models that generalize beyond a single dataset, such as FORECasT-BE will prove a useful tool when ones specialised to the context of interest are not available.

Predictions were more accurate at positions further away from the canonical window center for both existing and our new models. This is potentially due to increased measurement noise in central positions. While the Pearson's correlation between editing in replicates is consistent across the entire window, the mean squared error is much higher at central positions in all editors that we screened, reflecting larger absolute differences between replicate measurements (Supplementary Figure S1A–D). Together, these observations indicate that it is easier to rank potential bystander editing events away from editing window center, while both ranking and absolute rate prediction are relatively more difficult in the central region with more dynamic range.

Both *in vitro* genome engineering and *in silico* machine learning model building require making choices that can impact analyses and inferences. First, our data for cytosine editors was generated using lentiviral infection of the construct in K562 cells, while the adenine editors were screened using transient transfection of the editor and construct in HEK293T cells. Given the experimentally determined similarity between the two approaches (Supplementary Figure S1E), along with the confirmation of observed trends in other datasets, we do not think that this difference had a substantial effect on the results. Second, measures of gRNA efficacy such as DeepSpCas9 score, RuleSet2 score and observed editing rate in published Cas9 screens all had low correlation with base editing efficiency. However, the measures we chose to use were all generated for cutting Cas9, while base editors use a nicking Cas9. It is possible that this is a limiting factor in how informative these measures can be of editing rate, but is also the closest available match in the absence of large-scale measurements of nicking Cas9.

Using FORECasT-BE, we find that most pathogenic SNPs that could be reverted using cytosine base editors are expected to have more unintended edits than clean conversions, but identify 108 guides for C to T editing and 421 guides for A to G editing with promise to cleanly correct their targets. The editing of non-targeted cytosines presents a hurdle for clinical use of base editors. While the technology develops to address these issues, predictive models will remain essential to identify unintentional edits in advance, to potentially account for their effects (51), and to evaluate the pathogenicity of a guide when planning therapies.

As base editors are already used in genetic screens (52,53), have demonstrated feasibility in preclinical settings (54–57), and will likely soon advance into clinical trials, there is a need for better understanding of editing determinants and more accurate models of their effects. This work, and predictive outcome models in general, will be necessary to support the use of base editing in scientific and therapeutic applications.

## DATA AVAILABILITY

**Sequencing data:** European Nucleotide Archive (https://www.ebi.ac.uk/ena/browser/home) accession PRJEB12405, see Supplementary Table S4 for sample accessions.

**Processed data:** https://doi.org/10.6084/m9.figshare.14460039.

**Guides used in this study:** https://doi.org/10.6084/m9.figshare.14460138.

**FORECasT-BE code:** https://github.com/ananth-pallaseni/FORECasT-BE.

## Supplementary Material

gkac161_Supplemental_Files

## References

[1] Doudna J.A. , Charpentier E. The new frontier of genome engineering with CRISPR-Cas9. Science. 2014; 346:1258096. 25430774 10.1126/science.1258096

[2] Pickar-Oliver A. , Gersbach C.A. The next generation of CRISPR–Cas technologies and applications. Nat. Rev. Mol. Cell Biol. 2019; 20:490–507. 31147612 10.1038/s41580-019-0131-5 PMC7079207

[3] Behan F.M. , Iorio F., Picco G., Gonçalves E., Beaver C.M., Migliardi G., Santos R., Rao Y., Sassi F., Pinnelli M. et al. Prioritization of cancer therapeutic targets using CRISPR–Cas9 screens. Nature. 2019; 568:511–516. 30971826 10.1038/s41586-019-1103-9

[4] Min Y.-L. , Li H., Rodriguez-Caycedo C., Mireault A.A., Huang J., Shelton J.M., McAnally J.R., Amoasii L., Mammen P.P.A., Bassel-Duby R. et al. CRISPR-Cas9 corrects duchenne muscular dystrophy exon 44 deletion mutations in mice and human cells. Sci. Adv. 2019; 5:eaav4324. 30854433 10.1126/sciadv.aav4324 PMC6402849

[5] Park S.H. , Lee C.M., Dever D.P., Davis T.H., Camarena J., Srifa W., Zhang Y., Paikari A., Chang A.K., Porteus M.H. et al. Highly efficient editing of the β-globin gene in patient-derived hematopoietic stem and progenitor cells to treat sickle cell disease. Nucleic Acids Res. 2019; 47:7955–7972. 31147717 10.1093/nar/gkz475 PMC6735704

[6] Gilbert L.A. , Horlbeck M.A., Adamson B., Villalta J.E., Chen Y., Whitehead E.H., Guimaraes C., Panning B., Ploegh H.L., Bassik M.C. et al. Genome-scale CRISPR-mediated control of gene repression and activation. Cell. 2014; 159:647–661. 25307932 10.1016/j.cell.2014.09.029 PMC4253859

[7] Qi L.S. , Larson M.H., Gilbert L.A., Doudna J.A., Weissman J.S., Arkin A.P., Lim W.A. Repurposing CRISPR as an RNA-guided platform for sequence-specific control of gene expression. Cell. 2013; 152:1173–1183. 23452860 10.1016/j.cell.2013.02.022 PMC3664290

[8] Komor A.C. , Kim Y.B., Packer M.S., Zuris J.A., Liu D.R. Programmable editing of a target base in genomic DNA without double-stranded DNA cleavage. Nature. 2016; 533:420–424. 27096365 10.1038/nature17946 PMC4873371

[9] Gaudelli N.M. , Komor A.C., Rees H.A., Packer M.S., Badran A.H., Bryson D.I., Liu D.R. Programmable base editing of A•T to G•C in genomic DNA without DNA cleavage. Nature. 2017; 551:464–471. 29160308 10.1038/nature24644 PMC5726555

[10] Anzalone A.V. , Randolph P.B., Davis J.R., Sousa A.A., Koblan L.W., Levy J.M., Chen P.J., Wilson C., Newby G.A., Raguram A. et al. Search-and-replace genome editing without double-strand breaks or donor DNA. Nature. 2019; 576:149–157. 31634902 10.1038/s41586-019-1711-4 PMC6907074

[11] Ihry R.J. , Worringer K.A., Salick M.R., Frias E., Ho D., Theriault K., Kommineni S., Chen J., Sondey M., Ye C. et al. p53 inhibits CRISPR–Cas9 engineering in human pluripotent stem cells. Nat. Med. 2018; 24:939–946. 29892062 10.1038/s41591-018-0050-6

[12] Peets E.M. , Crepaldi L., Zhou Y., Allen F., Elmentaite R., Noell G., Turner G., Iyer V., Parts L. Minimized double guide RNA libraries enable scale-limited CRISPR/Cas9 screens. 2019; bioRxiv doi:29 November 2019, preprint: not peer reviewed 10.1101/859652.

[13] Anzalone A.V. , Koblan L.W., Liu D.R. Genome editing with CRISPR–Cas nucleases, base editors, transposases and prime editors. Nat. Biotechnol. 2020; 38:824–844. 32572269 10.1038/s41587-020-0561-9

[14] Thuronyi B.W. , Koblan L.W., Levy J.M., Yeh W.-H., Zheng C., Newby G.A., Wilson C., Bhaumik M., Shubina-Oleinik O., Holt J.R. et al. Continuous evolution of base editors with expanded target compatibility and improved activity. Nat. Biotechnol. 2019; 37:1070–1079. 31332326 10.1038/s41587-019-0193-0 PMC6728210

[15] Gehrke J.M. , Cervantes O., Clement M.K., Wu Y., Zeng J., Bauer D.E., Pinello L., Joung J.K. An APOBEC3A-Cas9 base editor with minimized bystander and off-target activities. Nat. Biotechnol. 2018; 36:977–982. 30059493 10.1038/nbt.4199 PMC6181770

[16] Kim Y.B. , Komor A.C., Levy J.M., Packer M.S., Zhao K.T., Liu D.R. Increasing the genome-targeting scope and precision of base editing with engineered Cas9-cytidine deaminase fusions. Nat. Biotechnol. 2017; 35:371–376. 28191901 10.1038/nbt.3803 PMC5388574

[17] Doman J.L. , Raguram A., Newby G.A., Liu D.R. Evaluation and minimization of Cas9-independent off-target DNA editing by cytosine base editors. Nat. Biotechnol. 2020; 38:620–628. 32042165 10.1038/s41587-020-0414-6 PMC7335424

[18] Gaudelli N.M. , Lam D.K., Rees H.A., Solá-Esteves N.M., Barrera L.A., Born D.A., Edwards A., Gehrke J.M., Lee S.-J., Liquori A.J. et al. Directed evolution of adenine base editors with increased activity and therapeutic application. Nat. Biotechnol. 2020; 38:892–900. 32284586 10.1038/s41587-020-0491-6

[19] Kurt I.C. , Zhou R., Iyer S., Garcia S.P., Miller B.R., Langner L.M., Grünewald J., Joung J.K. CRISPR C-to-G base editors for inducing targeted DNA transversions in human cells. Nat. Biotechnol. 2021; 39:41–46. 32690971 10.1038/s41587-020-0609-x PMC7854778

[20] Zhao D. , Li J., Li S., Xin X., Hu M., Price M.A., Rosser S.J., Bi C., Zhang X. Glycosylase base editors enable C-to-A and C-to-G base changes. Nat. Biotechnol. 2021; 39:35–40. 32690970 10.1038/s41587-020-0592-2

[21] Arbab M. , Shen M.W., Mok B., Wilson C., Matuszek Ż., Cassa C.A., Liu D.R. Determinants of base editing outcomes from target library analysis and machine learning. Cell. 2020; 182:463–480. 32533916 10.1016/j.cell.2020.05.037 PMC7384975

[22] Song M. , Kim H.K., Lee S., Kim Y., Seo S.-Y., Park J., Choi J.W., Jang H., Shin J.H., Min S. et al. Sequence-specific prediction of the efficiencies of adenine and cytosine base editors. Nat. Biotechnol. 2020; 38:1037–1043. 32632303 10.1038/s41587-020-0573-5

[23] Zuo E. , Sun Y., Wei W., Yuan T., Ying W., Sun H., Yuan L., Steinmetz L.M., Li Y., Yang H. Cytosine base editor generates substantial off-target single-nucleotide variants in mouse embryos. Science. 2019; 364:289–292. 30819928 10.1126/science.aav9973 PMC7301308

[24] Kim D. , Lim K., Kim S.-T., Yoon S.-H., Kim K., Ryu S.-M., Kim J.-S Genome-wide target specificities of CRISPR RNA-guided programmable deaminases. Nat. Biotechnol. 2017; 35:475–480. 28398345 10.1038/nbt.3852

[25] Jin S. , Zong Y., Gao Q., Zhu Z., Wang Y., Qin P., Liang C., Wang D., Qiu J.-L., Zhang F. et al. Cytosine, but not adenine, base editors induce genome-wide off-target mutations in rice. Science. 2019; 364:292–295. 30819931 10.1126/science.aaw7166

[26] Rees H.A. , Liu D.R. Publisher correction: base editing: precision chemistry on the genome and transcriptome of living cells. Nat. Rev. Genet. 2018; 19:801. 10.1038/s41576-018-0068-0 30341440

[27] Shi K. , Carpenter M.A., Banerjee S., Shaban N.M., Kurahashi K., Salamango D.J., McCann J.L., Starrett G.J., Duffy J.V., Demir Ö. et al. Structural basis for targeted DNA cytosine deamination and mutagenesis by APOBEC3A and APOBEC3B. Nat. Struct. Mol. Biol. 2016; 24:131–139. 27991903 10.1038/nsmb.3344 PMC5296220

[28] Saraconi G. , Severi F., Sala C., Mattiuz G., Conticello S.G. The RNA editing enzyme APOBEC1 induces somatic mutations and a compatible mutational signature is present in esophageal adenocarcinomas. Genome Biol. 2014; 15:417. 25085003 10.1186/s13059-014-0417-z PMC4144122

[29] Sakata R.C. , Ishiguro S., Mori H., Tanaka M., Tatsuno K., Ueda H., Yamamoto S., Seki M., Masuyama N., Nishida K. et al. Base editors for simultaneous introduction of C-to-T and A-to-G mutations. Nat. Biotechnol. 2020; 38:865–869. 32483365 10.1038/s41587-020-0509-0

[30] Zafra M.P. , Schatoff E.M., Katti A., Foronda M., Breinig M., Schweitzer A.Y., Simon A., Han T., Goswami S., Montgomery E. et al. Optimized base editors enable efficient editing in cells, organoids and mice. Nat. Biotechnol. 2018; 36:888–893. 29969439 10.1038/nbt.4194 PMC6130889

[31] Richter M.F. , Zhao K.T., Eton E., Lapinaite A., Newby G.A., Thuronyi B.W., Wilson C., Koblan L.W., Zeng J., Bauer D.E. et al. Phage-assisted evolution of an adenine base editor with improved cas domain compatibility and activity. Nat. Biotechnol. 2020; 38:883–891. 32433547 10.1038/s41587-020-0453-z PMC7357821

[32] Allen F. , Crepaldi L., Alsinet C., Strong A.J., Kleshchevnikov V., De Angeli P., Páleníková P., Khodak A., Kiselev V., Kosicki M. et al. Predicting the mutations generated by repair of Cas9-induced double-strand breaks. Nat. Biotechnol. 2018; 37:64–72. 10.1038/nbt.4317 PMC6949135 30480667

[33] Tzelepis K. , Koike-Yusa H., De Braekeleer E., Li Y., Metzakopian E., Dovey O.M., Mupo A., Grinkevich V., Li M., Mazan M. et al. A CRISPR dropout screen identifies genetic vulnerabilities and therapeutic targets in acute myeloid leukemia. Cell Rep. 2016; 17:1193–1205. 27760321 10.1016/j.celrep.2016.09.079 PMC5081405

[34] Pirona A.C. , Oktriani R., Boettcher M., Hoheisel J.D. Process for an efficient lentiviral cell transduction. Biol Methods Protoc. 2020; 5:bpaa005. 32395634 10.1093/biomethods/bpaa005 PMC7200879

[35] Nishida K. , Arazoe T., Yachie N., Banno S., Kakimoto M., Tabata M., Mochizuki M., Miyabe A., Araki M., Hara K.Y. et al. Targeted nucleotide editing using hybrid prokaryotic and vertebrate adaptive immune systems. Science. 2016; 353:aaf8729. 27492474 10.1126/science.aaf8729

[36] Sanjana N.E. , Shalem O., Zhang F. Improved vectors and genome-wide libraries for CRISPR screening. Nat. Methods. 2014; 11:783–784. 25075903 10.1038/nmeth.3047 PMC4486245

[37] Clement K. , Rees H., Canver M.C., Gehrke J.M., Farouni R., Hsu J.Y., Cole M.A., Liu D.R., Joung J.K., Bauer D.E. et al. CRISPResso2 provides accurate and rapid genome editing sequence analysis. Nat. Biotechnol. 2019; 37:224–226. 30809026 10.1038/s41587-019-0032-3 PMC6533916

[38] Cock P.J.A. , Antao T., Chang J.T., Chapman B.A., Cox C.J., Dalke A., Friedberg I., Hamelryck T., Kauff F., Wilczynski B. et al. Biopython: freely available python tools for computational molecular biology and bioinformatics. Bioinformatics. 2009; 25:1422–1423. 19304878 10.1093/bioinformatics/btp163 PMC2682512

[39] Doench J.G. , Fusi N., Sullender M., Hegde M., Vaimberg E.W., Donovan K.F., Smith I., Tothova Z., Wilen C., Orchard R. et al. Optimized sgRNA design to maximize activity and minimize off-target effects of CRISPR-Cas9. Nat. Biotechnol. 2016; 34:184–191. 26780180 10.1038/nbt.3437 PMC4744125

[40] Pedregosa F. , Varoquaux G., Gramfort A., Michel V., Thirion B., Grisel O., Blondel M., Prettenhofer P., Weiss R., Dubourg V. et al. Scikit-learn: machine learning in python. J. Mach. Learn. Res. 2011; 12:2825–2830.

[41] Paszke A. , Gross S., Massa F., Lerer A., Bradbury J., Chanan G., Killeen T., Lin Z., Gimelshein N., Antiga L. et al. Wallach H. , Larochelle H., Beygelzimer A., d’Alché-Buc F., Fox E., Garnett R. PyTorch: an imperative style, high-performance deep learning library. Advances in Neural Information Processing Systems. 2019; 32:Curran Associates, Inc.

[42] Komor A.C. , Zhao K.T., Packer M.S., Gaudelli N.M., Waterbury A.L., Koblan L.W., Kim Y.B., Badran A.H., Liu D.R. Improved base excision repair inhibition and bacteriophage mu gam protein yields C:G-to-T:A base editors with higher efficiency and product purity. Sci. Adv. 2017; 3:eaao4774. 28875174 10.1126/sciadv.aao4774 PMC5576876

[43] Rabinowitz R. , Abadi S., Almog S., Offen D Prediction of synonymous corrections by the BE-FF computational tool expands the targeting scope of base editing. Nucleic Acids Res. 2020; 48:W340–W347. 32255179 10.1093/nar/gkaa215 PMC7319459

[44] Wolf J. , Gerber A.P., Keller W. tadA, an essential tRNA-specific adenosine deaminase from escherichia coli. EMBO J. 2002; 21:3841–3851. 12110595 10.1093/emboj/cdf362 PMC126108

[45] Koblan L.W. , Arbab M., Shen M.W., Hussmann J.A., Anzalone A.V., Doman J.L., Newby G.A., Yang D., Mok B., Replogle J.M. et al. Efficient C•G-to-G•C base editors developed using CRISPRi screens, target-library analysis, and machine learning. Nat. Biotechnol. 2021; 39:1414–1425. 34183861 10.1038/s41587-021-00938-z PMC8985520

[46] Chen L. , Park J.E., Paa P., Rajakumar P.D., Prekop H.-T., Chew Y.T., Manivannan S.N., Chew W.L. Programmable C:G to G:C genome editing with CRISPR-Cas9-directed base excision repair proteins. Nat. Commun. 2021; 12:1384. 33654077 10.1038/s41467-021-21559-9 PMC7925527

[47] Yuan T. , Yan N., Fei T., Zheng J., Meng J., Li N., Liu J., Zhang H., Xie L., Ying W. et al. Optimization of C-to-G base editors with sequence context preference predictable by machine learning methods. Nat. Commun. 2021; 12:4902. 34385461 10.1038/s41467-021-25217-y PMC8361092

[48] Kim H.K. , Kim Y., Lee S., Min S., Bae J.Y., Choi J.W., Park J., Jung D., Yoon S., Kim H.H. SpCas9 activity prediction by deepspcas9, a deep learning–based model with high generalization performance. Sci. Adv. 2019; 5:eaax9249. 31723604 10.1126/sciadv.aax9249 PMC6834390

[49] Iijima O. , Fukano H., Takahashi H., Shirai M., Suzuki Y. A purine at+ 2 rather than+ 1 adjacent to the human U6 promoter is required to prepare effective short hairpin RNAs. Biochem. Biophys. Res. Commun. 2006; 350:809–817. 17045573 10.1016/j.bbrc.2006.08.187

[50] Friedman J.H. Greedy function approximation: a gradient boosting machine. Ann. Stat. 2001; 29:1189–1232.

[51] Huang C. , Li G., Wu J., Liang J., Wang X. Identification of pathogenic variants in cancer genes using base editing screens with editing efficiency correction. Genome Biol. 2021; 22:80. 33691754 10.1186/s13059-021-02305-2 PMC7945310

[52] Hanna R.E. , Hegde M., Fagre C.R., DeWeirdt P.C., Sangree A.K., Szegletes Z., Griffith A., Feeley M.N., Sanson K.R., Baidi Y. et al. Massively parallel assessment of human variants with base editor screens. Cell. 2021; 184:1064–1080. 33606977 10.1016/j.cell.2021.01.012

[53] Sangree A.K. , Griffith A.L., Szegletes Z.M., Roy P., DeWeirdt P.C., Hegde M., McGee A.V., Hanna R.E., Doench J.G. Benchmarking of spcas9 variants enables deeper base editor screens of BRCA1 and BCL2. 2021; bioRxiv doi:18 August 2021, preprint: not peer reviewed 10.1101/2021.08.18.456848. PMC8921519 35288574

[54] Koblan L.W. , Erdos M.R., Wilson C., Cabral W.A., Levy J.M., Xiong Z.-M., Tavarez U.L., Davison L.M., Gete Y.G., Mao X. et al. In vivo base editing rescues hutchinson–gilford progeria syndrome in mice. Nature. 2021; 589:608–614. 33408413 10.1038/s41586-020-03086-7 PMC7872200

[55] Lin L. , Rybak A.P., Rinaldi C., Yen J., Fu Y., Akrawi E., Smith S., Haskett S.J., Sanchez M.E., Poh Y.-C. et al. Complementary base editing approaches for the treatment of sickle cell disease and beta thalassemia. Blood. 2019; 134:3352–3352.

[56] Levy J.M. , Yeh W.-H., Pendse N., Davis J.R., Hennessey E., Butcher R., Koblan L.W., Comander J., Liu Q., Liu D.R. Cytosine and adenine base editing of the brain, liver, retina, heart and skeletal muscle of mice via adeno-associated viruses. Nat. Biomed. Eng. 2020; 4:97–110. 31937940 10.1038/s41551-019-0501-5 PMC6980783

[57] Banskota S. , Raguram A., Suh S., Du S.W., Davis J.R., Choi E.H., Wang X., Nielsen S.C., Newby G.A., Randolph P.B. et al. Engineered virus-like particles for efficient in vivo delivery of therapeutic proteins. Cell. 2022; 185:250–265. 35021064 10.1016/j.cell.2021.12.021 PMC8809250

