# Supplementary material for: Predicting base editing outcomes using position-specific sequence determinants
Source: Nucleic Acids Res. 2022 Mar 14;50(6):3551–64. doi: 10.1093/nar/gkac161 (PMC8989541; doi:10.1093/nar/gkac161)
Supplement: gkac161_Supplemental_Files [file gkac161_supplemental_files.zip › Base Editor Manuscript Supplementaries NAR.pdf]

Predicting base editing outcomes using position-specific sequence determinants

Supplementary Information

Figure S1A

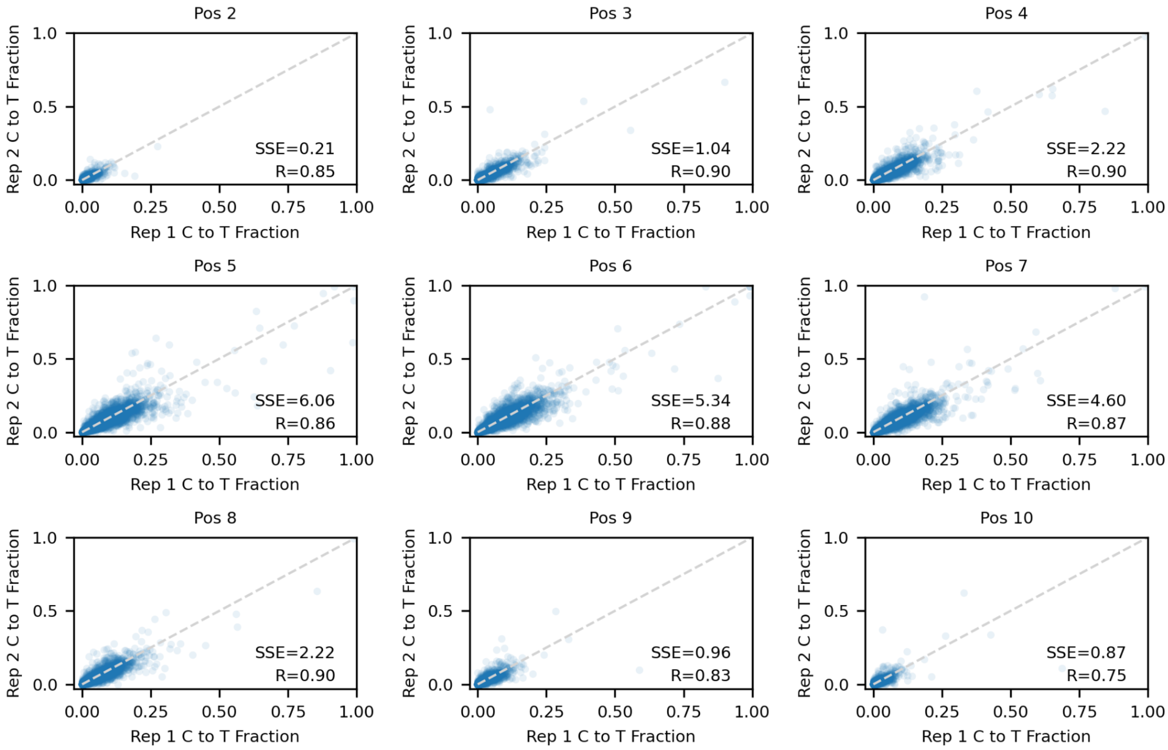

Base editor efficacy is reproducible between replicates in BE4. Fraction of reads with C to T edits in replicate 1 (x-axis) is strongly correlated with the fraction of reads with C to T edits in replicate 2 (y-axis) for cytosines in different positions of the target sequence (panels) across different targets (markers). Labels: Pearson's R (R) and sum of squared error (SSE) between measured and predicted scores. Dashed line: x=y.

Figure S1B

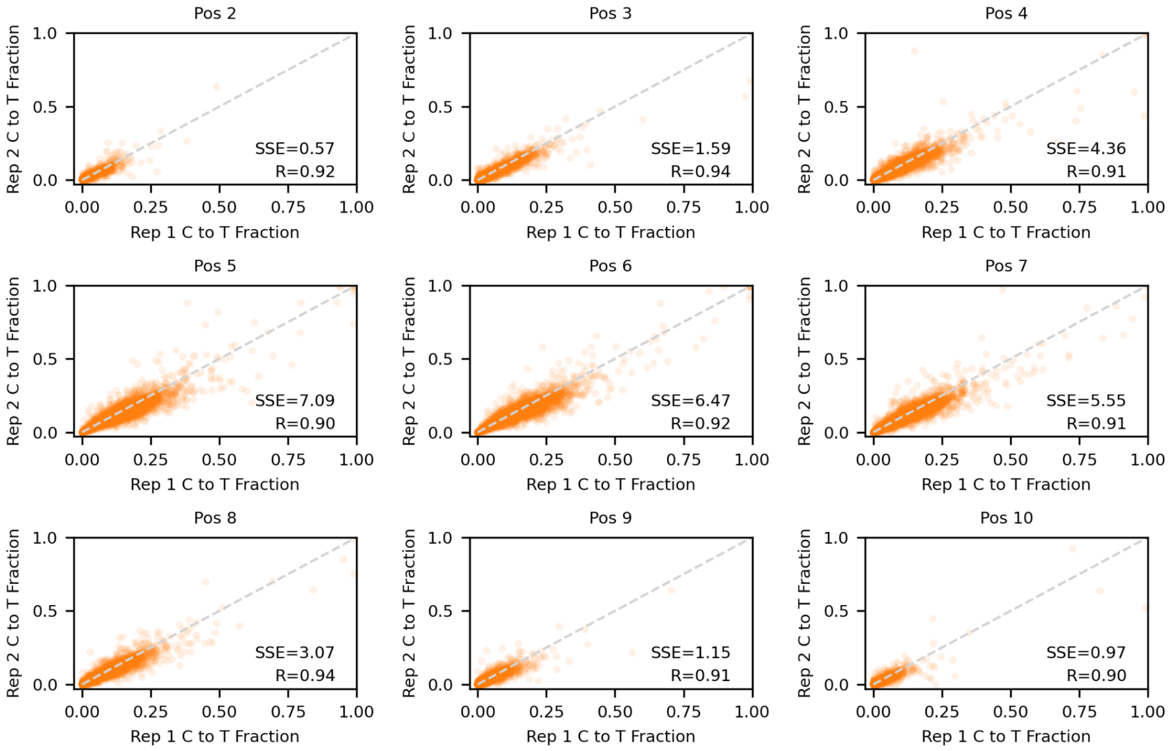

Base editor efficacy is reproducible between replicates in FNLS. Fraction of reads with C to T edits in replicate 1 (x-axis) is strongly correlated with the fraction of reads with C to T edits in replicate 2 (y-axis) for cytosines in different positions of the target sequence (panels) across different targets (markers). Labels: Pearson's R (R) and sum of squared error (SSE) between measured and predicted scores. Dashed line: x=y.

Figure S1C

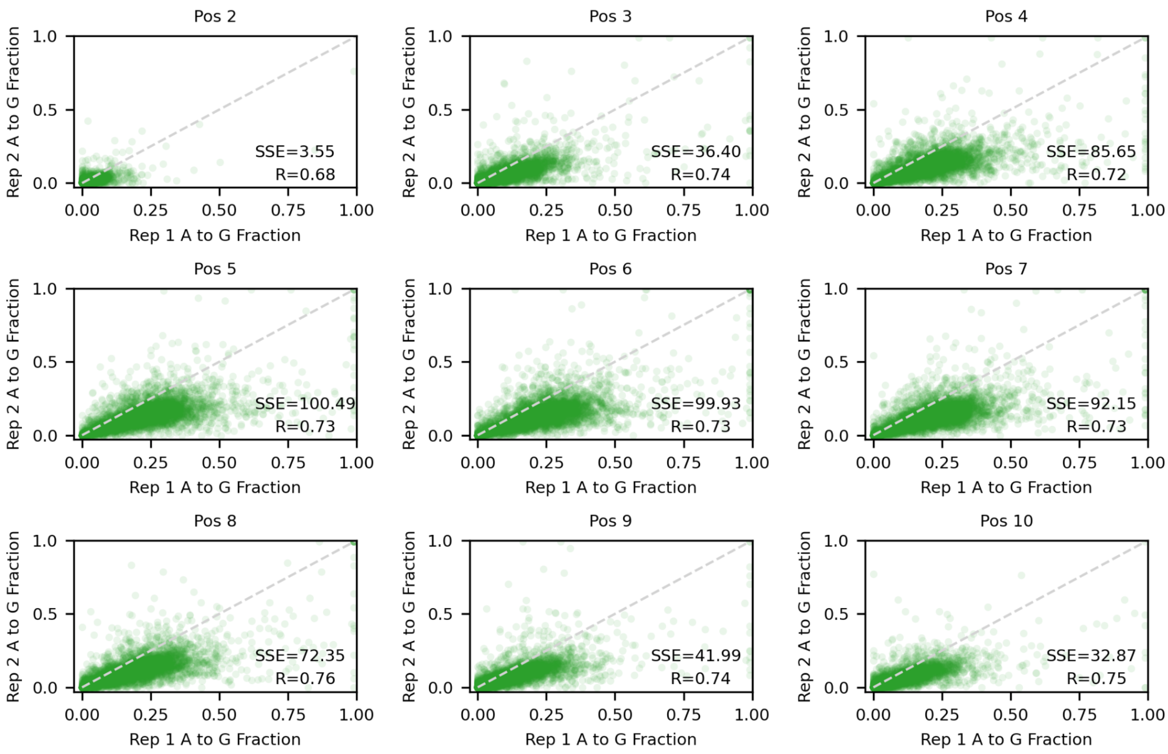

Base editor efficacy is reproducible between replicates in ABE8e. Fraction of reads with A to G edits in replicate 1 (x-axis) is strongly correlated with the fraction of reads with A to G edits in replicate 2 (y-axis) for adenines in different positions of the target sequence (panels) across different targets (markers). Labels: Pearson's R (R) and sum of squared error (SSE) between measured and predicted scores. Dashed line: x=y.

Figure S1D

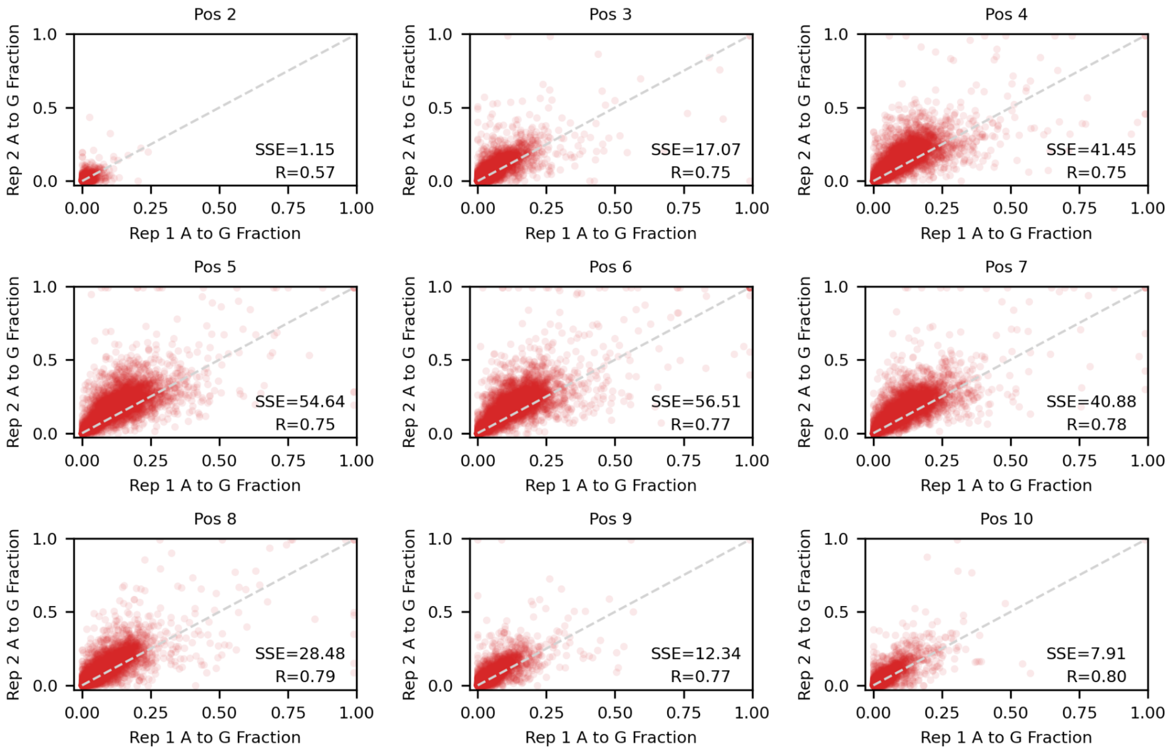

Base editor efficacy is reproducible between replicates in ABE20m. Fraction of reads with A to G edits in replicate 1 (x-axis) is strongly correlated with the fraction of reads with A to G edits in replicate 2 (y-axis) for adenines in different positions of the target sequence (panels) across different targets (markers). Labels: Pearson's R (R) and sum of squared error (SSE) between measured and predicted scores. Dashed line: x=y.

Figure S1E

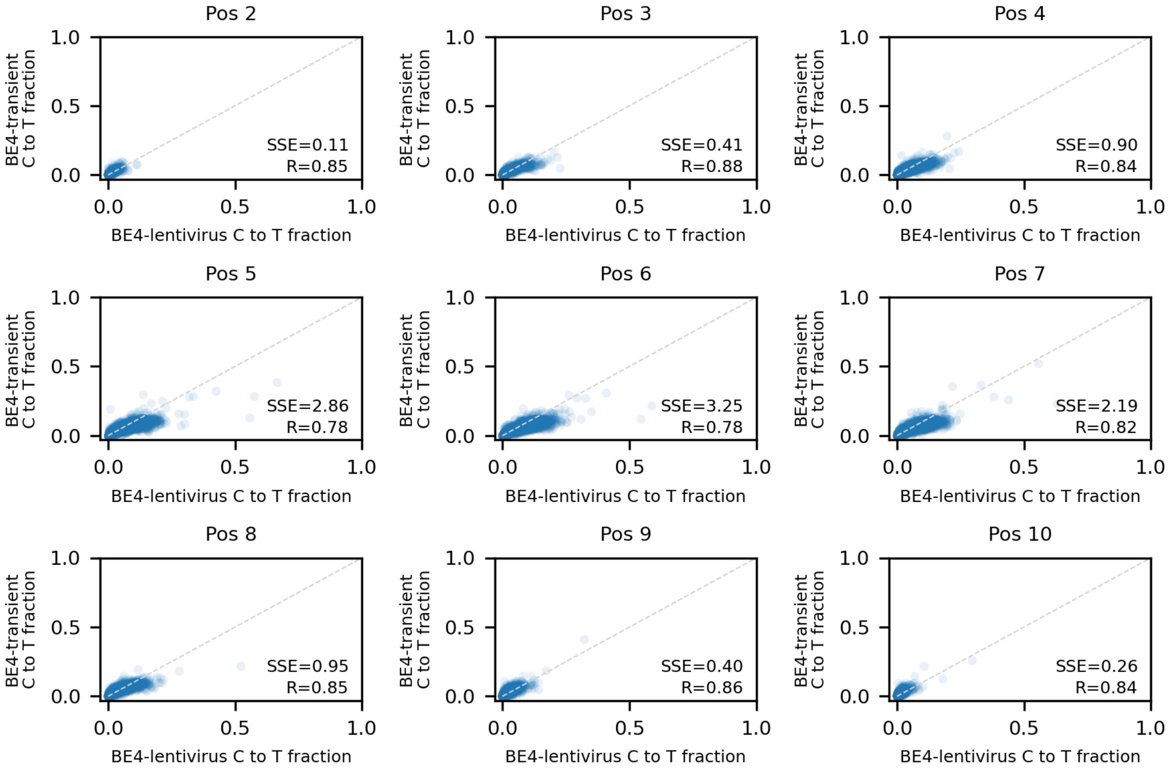

Base editor efficacy is reproducible between screens performed using different methodologies. Fraction of reads with C to T edits in BE4 screened using lentivirus (x-axis) is strongly correlated with the fraction of reads with C to T edits in BE4 screened using transient transfection (y-axis) for cytosines in different positions of the target sequence (panels) across different targets (markers). Dashed line: x=y.

Figure S1F

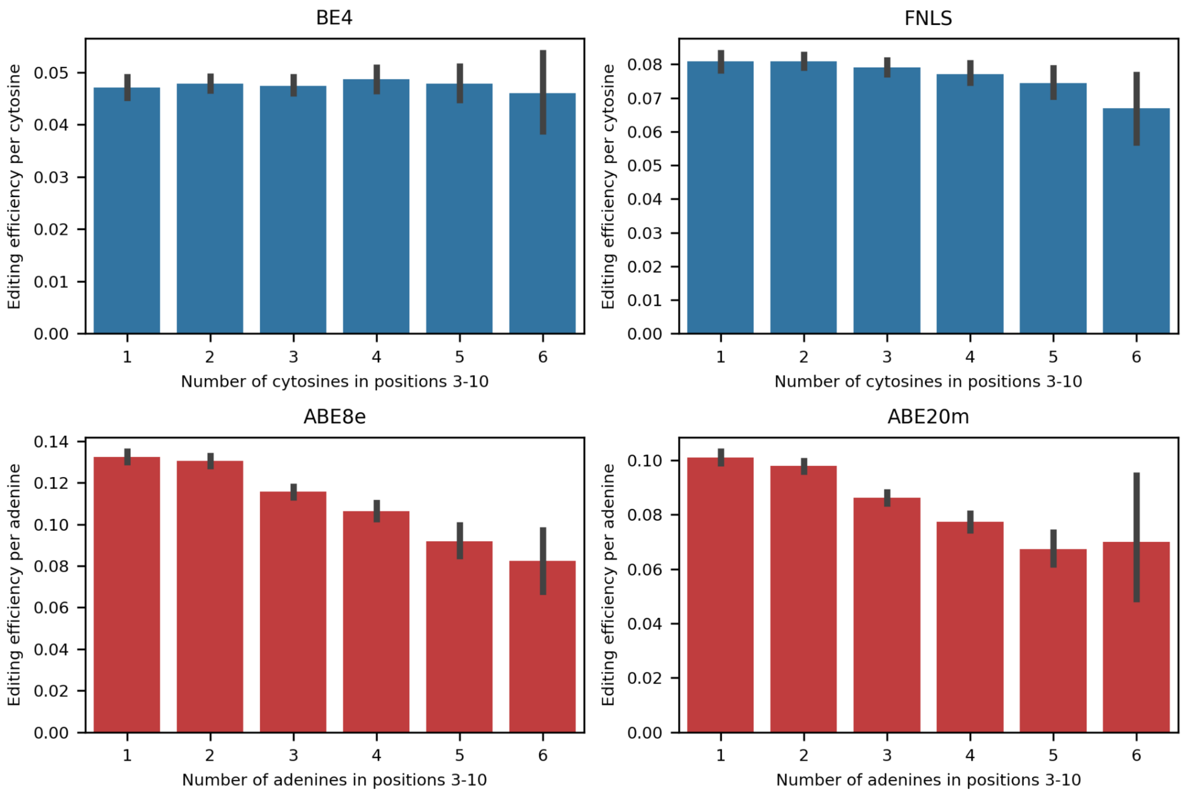

Editing efficiency per number of targetable bases (y-axis) for increasing number of targetable bases in the editing window (x-axis) for BE4 (top left), FNLS (top right), ABE8e (bottom left) and ABE20m (bottom right). Error bars: 95% confidence intervals from 1000 bootstrap samples.

Figure S1G

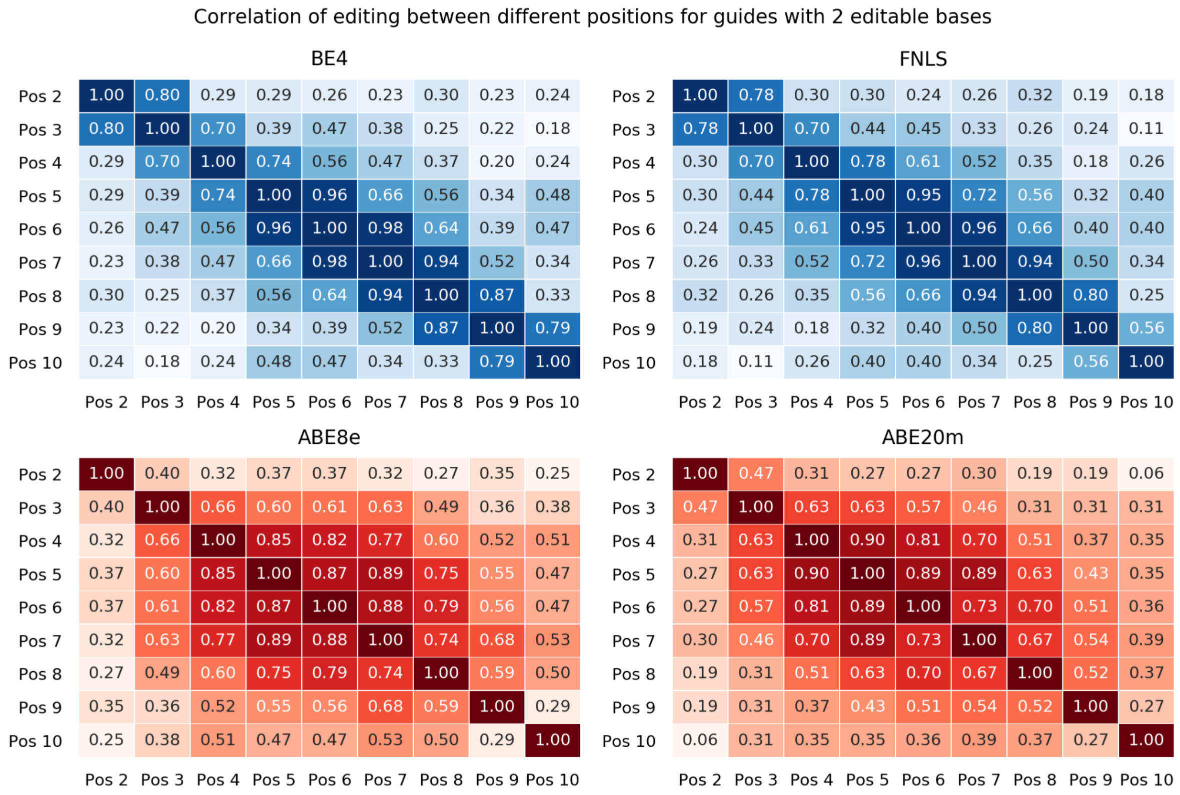

Editing at nearby positions is correlated. Pearson’s R between editing at one position (x-axis) and another position (y-axis) for guides with 2 targetable bases in positions 2-10 for BE4 (top left), FNLS (top right), ABE8e (bottom left) and ABE20m (bottom right).

Figure S1H

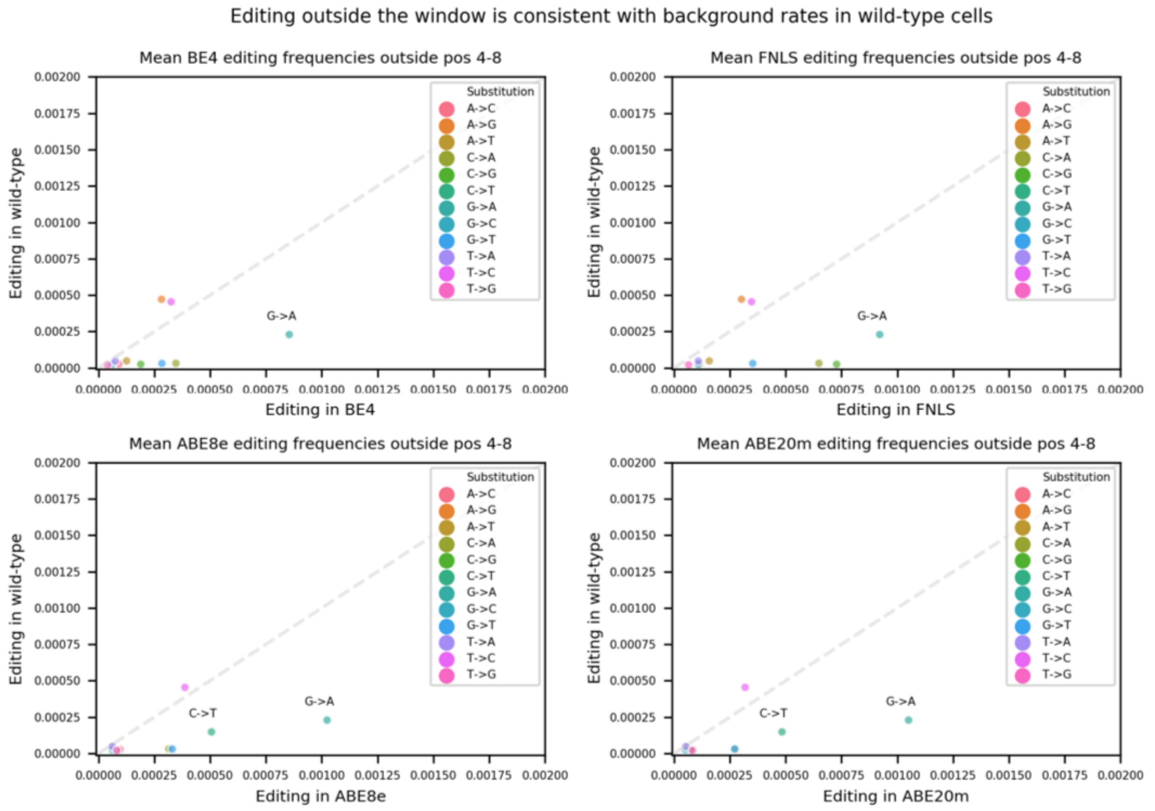

Unintentional substitutions (non C to T in cytosine editors and non A to G in adenine editors) outside of the target window occur at background rates, as measured in wild-type cells without base editors. Editing rate outside positions 4 to 8 in the target for wild type cells (y-axis) and base editor endowed cells (x-axis) for BE4 (top left), FNLS (top right), ABE8e (bottom left) and ABE20m (bottom right) for different substitution types (colors). Dashed line: y=x. Intended edits (C to T in the top panels, and A to G in the bottom panels) are omitted.

Figure S1I

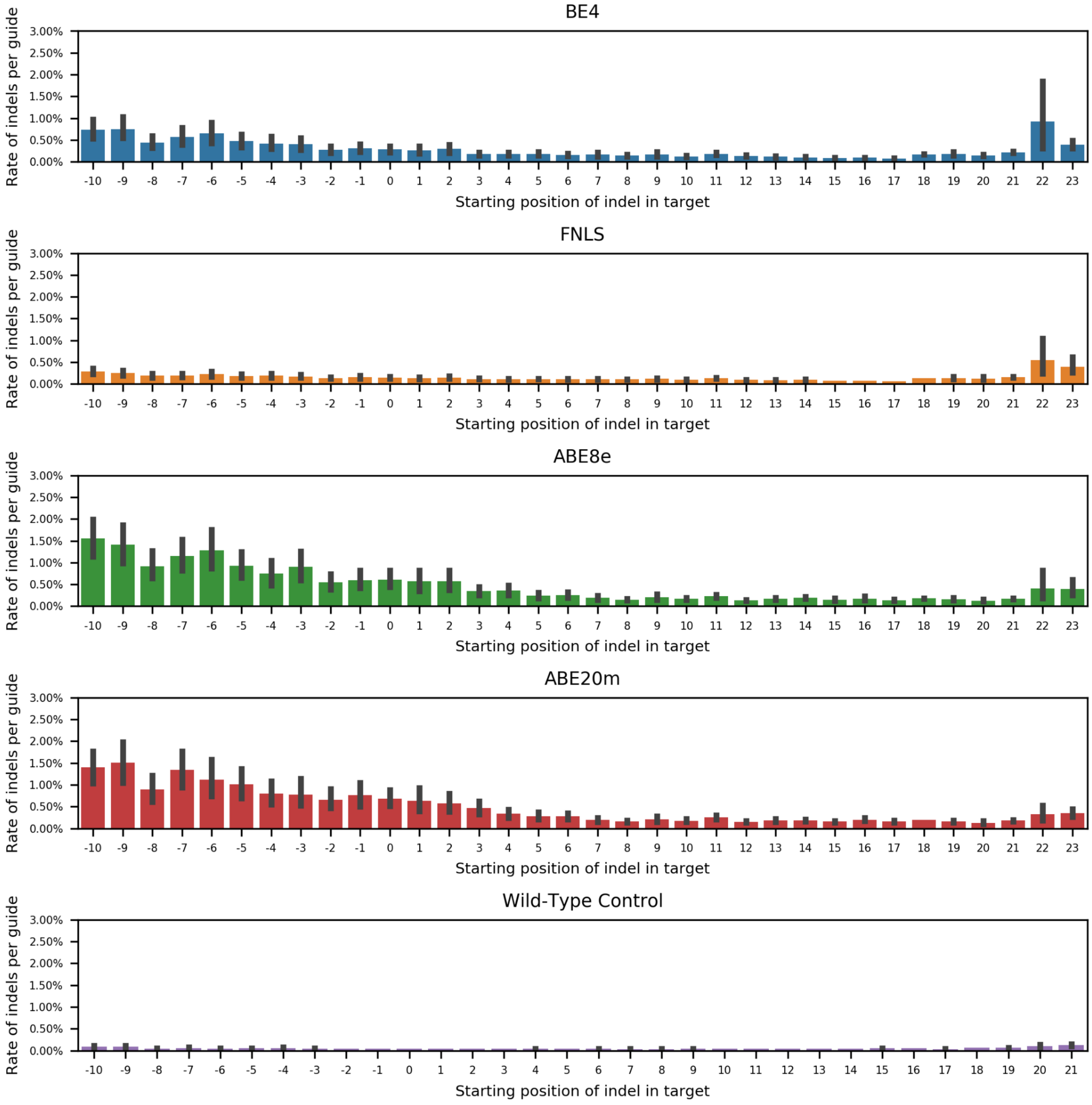

Insertions and deletion frequencies are low. Median frequency of indels per guide (y-axis) at each position in the target sequence (x-axis) for BE4 (top row), FNLS (second row), ABE8e (thrid row), ABE20m (fourth row) and cells without editors (bottom row). Error bars: 95% bootstrap confidence intervals from 1000 samples.

Figure S1J

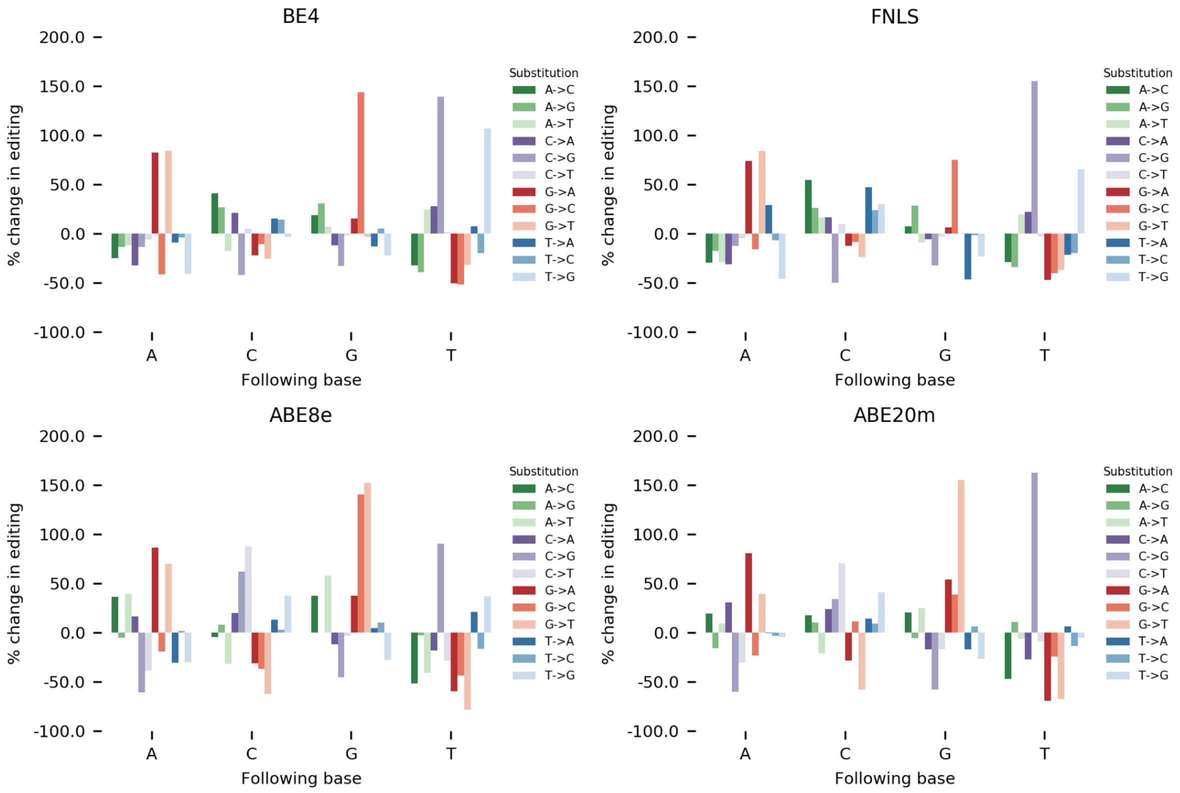

The influence of the base following the edited cytosine/adenine. Percentage change in editing rate (y-axis) when the target base is followed by a certain base (x-axis) versus all other bases for BE4 (top left), FNLS (top right), ABE8e (bottom left) and ABE20m (bottom right). Colors: substitutions.

Figure S1K

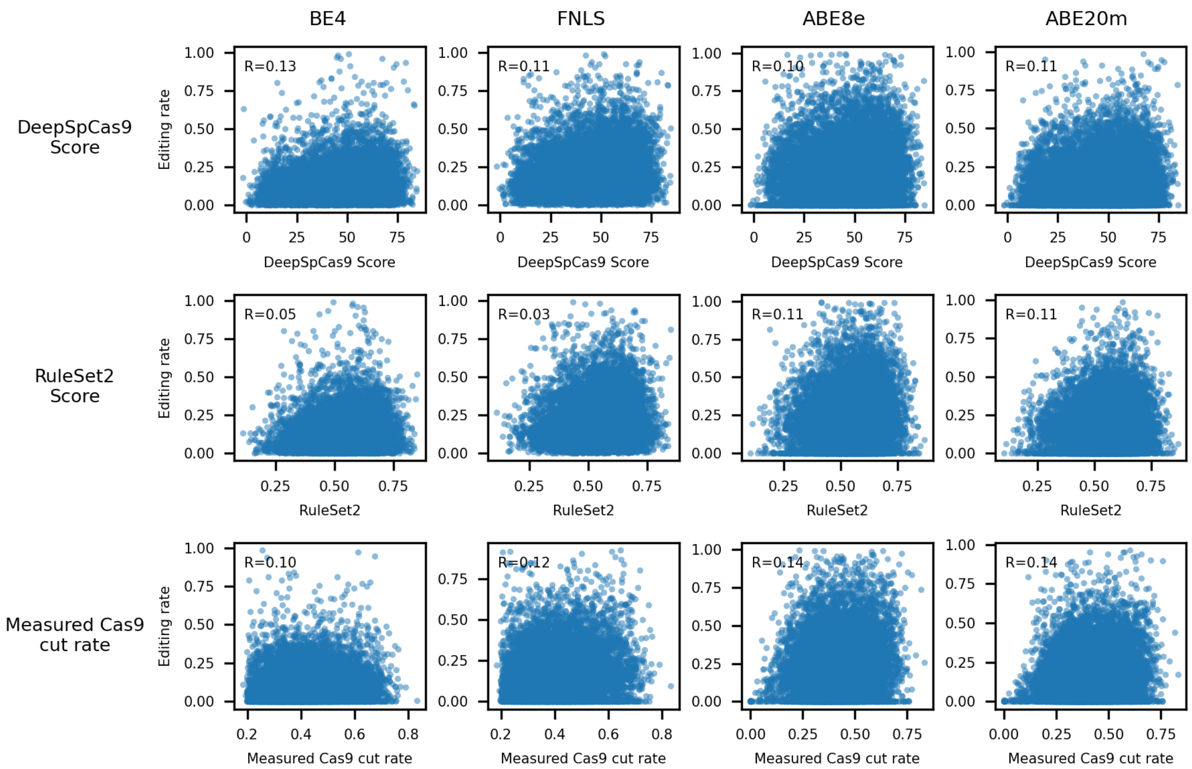

Metrics of guide RNA efficacy are weakly correlated with base editing. Total editing rate (y-axis) and DeepSpCas9 score (x-axis, top row), RuleSet2 score (x-axis, middle row) or measured Cas9 cut rate (x-axis, bottom row) for guide RNAs (markers) screened in all editors (columns). Label: Pearson's R between total editing rate and gRNA metric.

Figure S1L

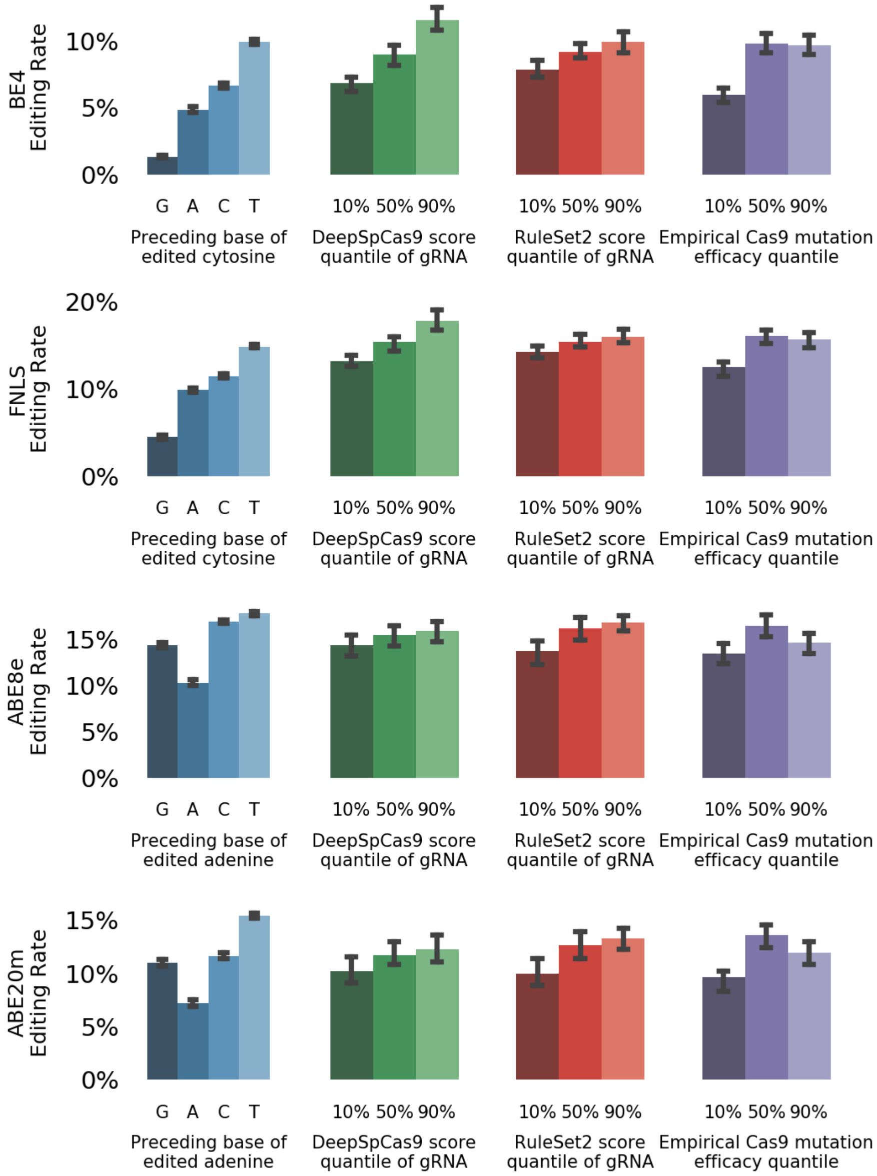

Cas9 gRNA features are informative of base editing rate. Median base editing rate (y-axis) is influenced by preceding base (blue bars), DeepSpCas9 score decile (green bars), RuleSet2 scores (red bars) and empirical Cas9 mutation efficacy (purple bars). Plots shown for BE4 (top row), FNLS (second row), ABE8e (third row) and ABE20m (bottom row). Error bars: 95% confidence intervals from 1000 bootstrap samples.

Figure S2A

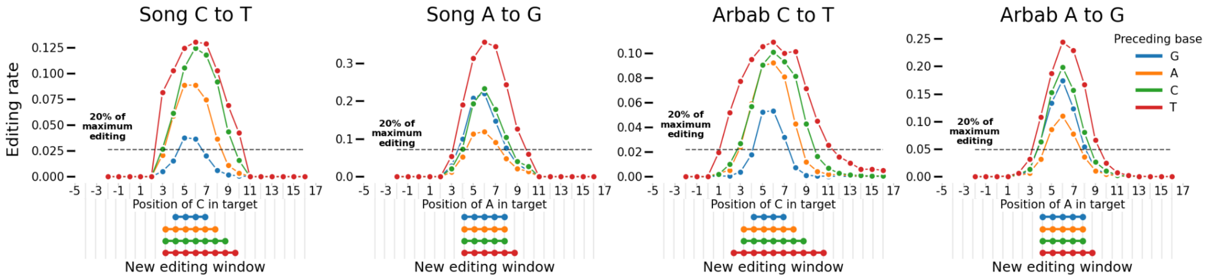

Window of editing changes depending on the base preceding the edited base in other studies. Median editing rate (y-axis) of adenines or cytosines at positions -5 to 17 in the target (x-axis) for each preceding base type (colors) in datasets from Song et al (left) and Arbab et al (right) for each editor type (panels). Black dashed line: 20% of the maximum editing rate at any position for all preceding bases. Linked dots: positions at which editing is above 20% of the maximum.

Figure S2B

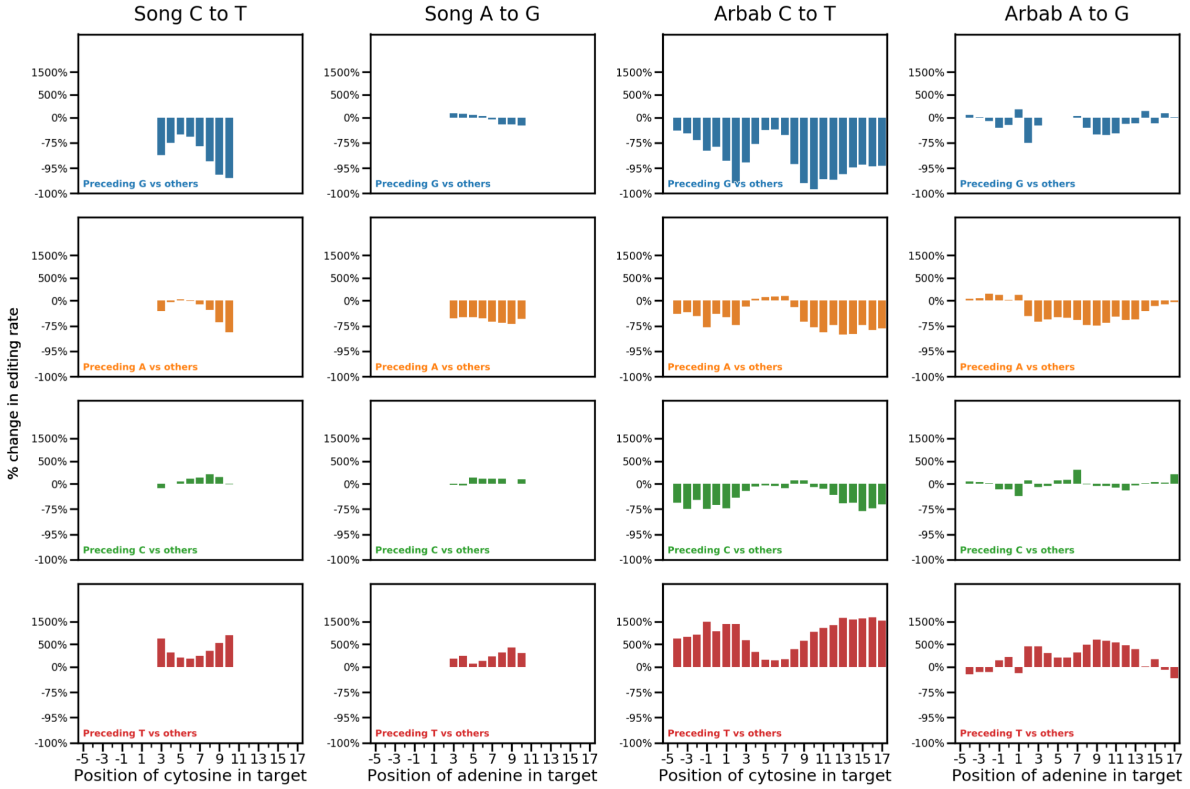

Position-dependent effect of the preceding base in Arbab and Song datasets for each type of editor (columns). Percentage change in editing rate (y-axis) from having a certain base preceding the cytosine (colors) compared to all other bases at different positions in the target sequence (x-axis).

Figure S2C

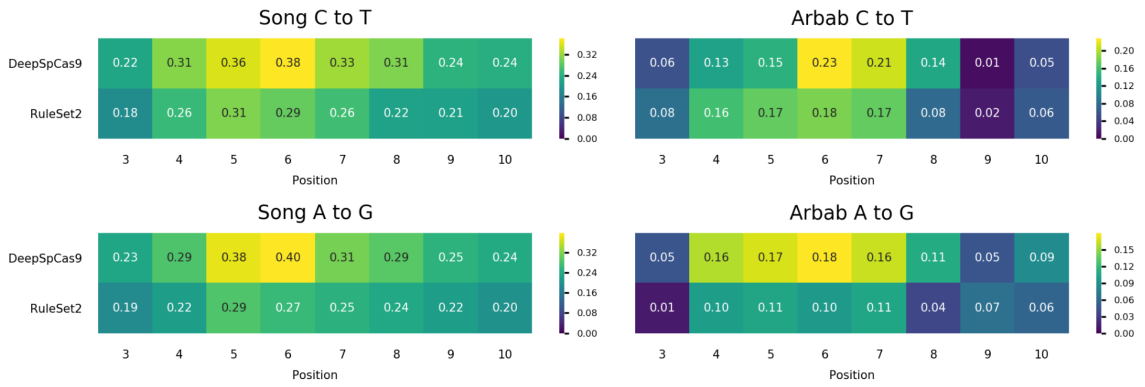

Correlation between gRNA quality and editing rate depend on the position in Song and Arbab datasets for each type of editor (panels). Pearson's R (color) between measures of gRNA quality (y-axis) and the position of the cytosine or adenine in the target sequence (x-axis).

Figure S2D

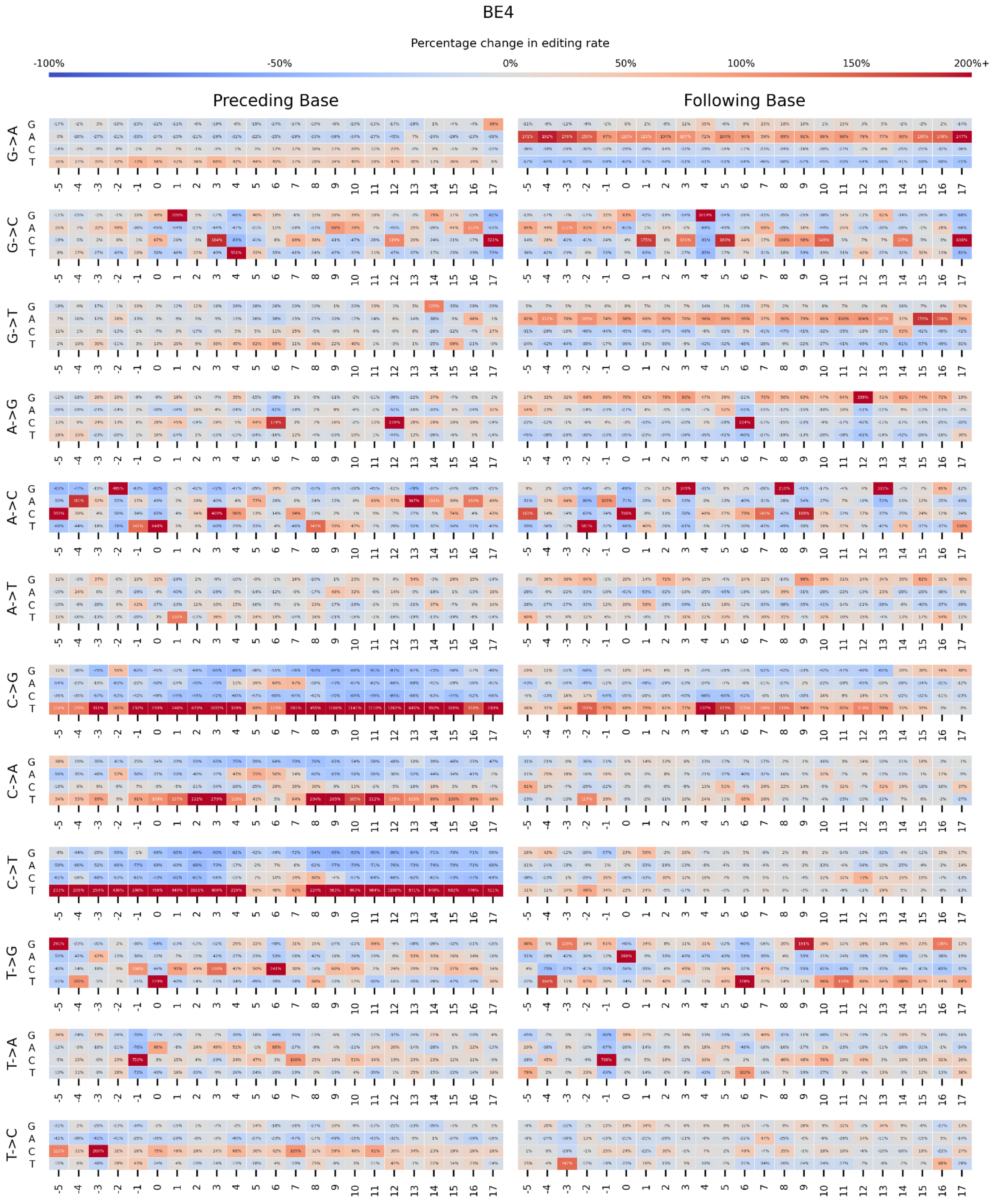

Position-dependent effect of the preceding and following base in BE4. Percentage change in editing rate (color) depending on the identity of the base (y-axis) preceding (left) or following (right) the target base in the sequence (x-axis) for each substitution type (rows).

Figure S2E

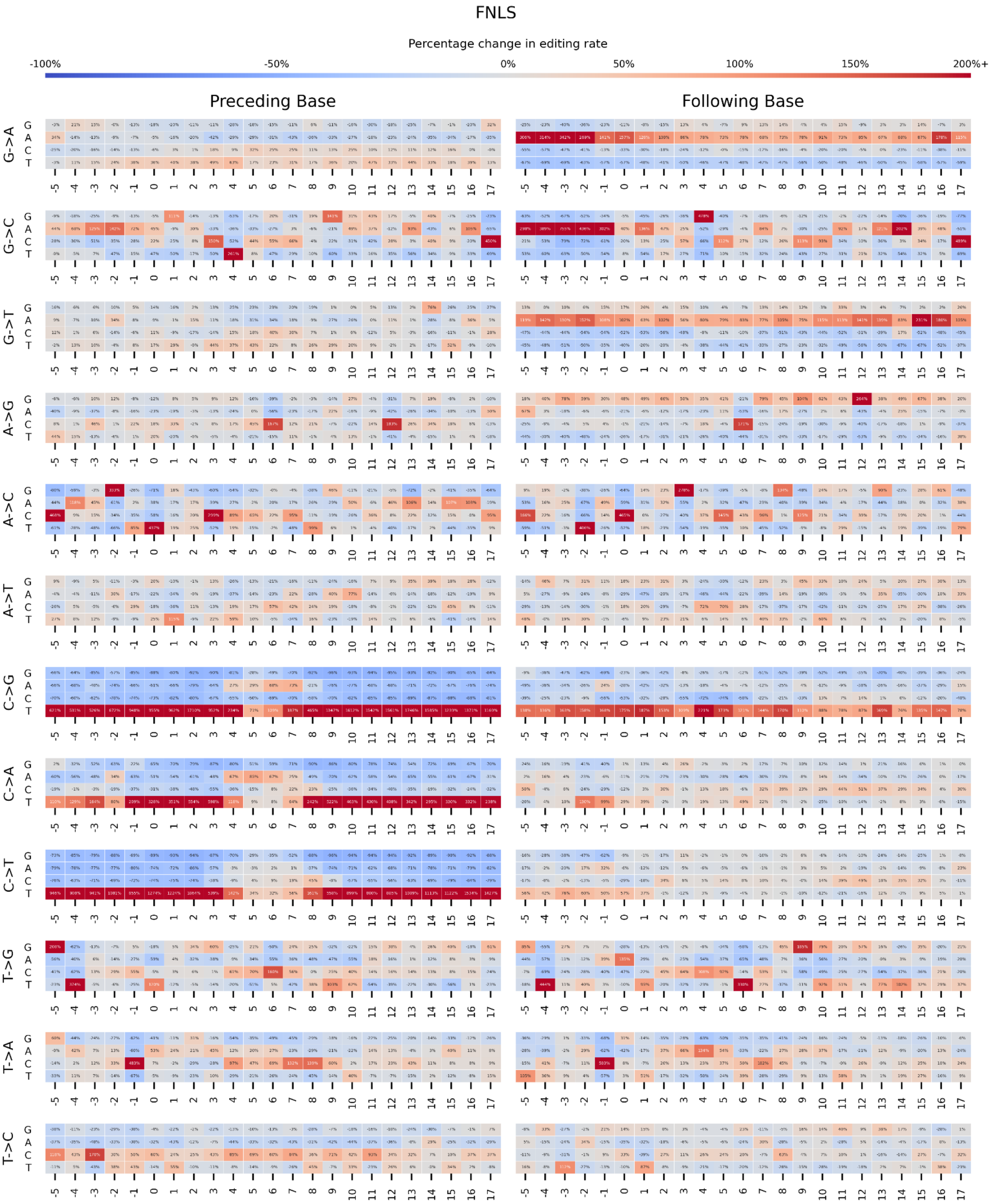

Position-dependent effect of the preceding and following base in FNLS. Percentage change in editing rate (color) depending on the identity of the base (y-axis) preceding (left) or following (right) the target base in the sequence (x-axis) for each substitution type (rows).

Figure S2F

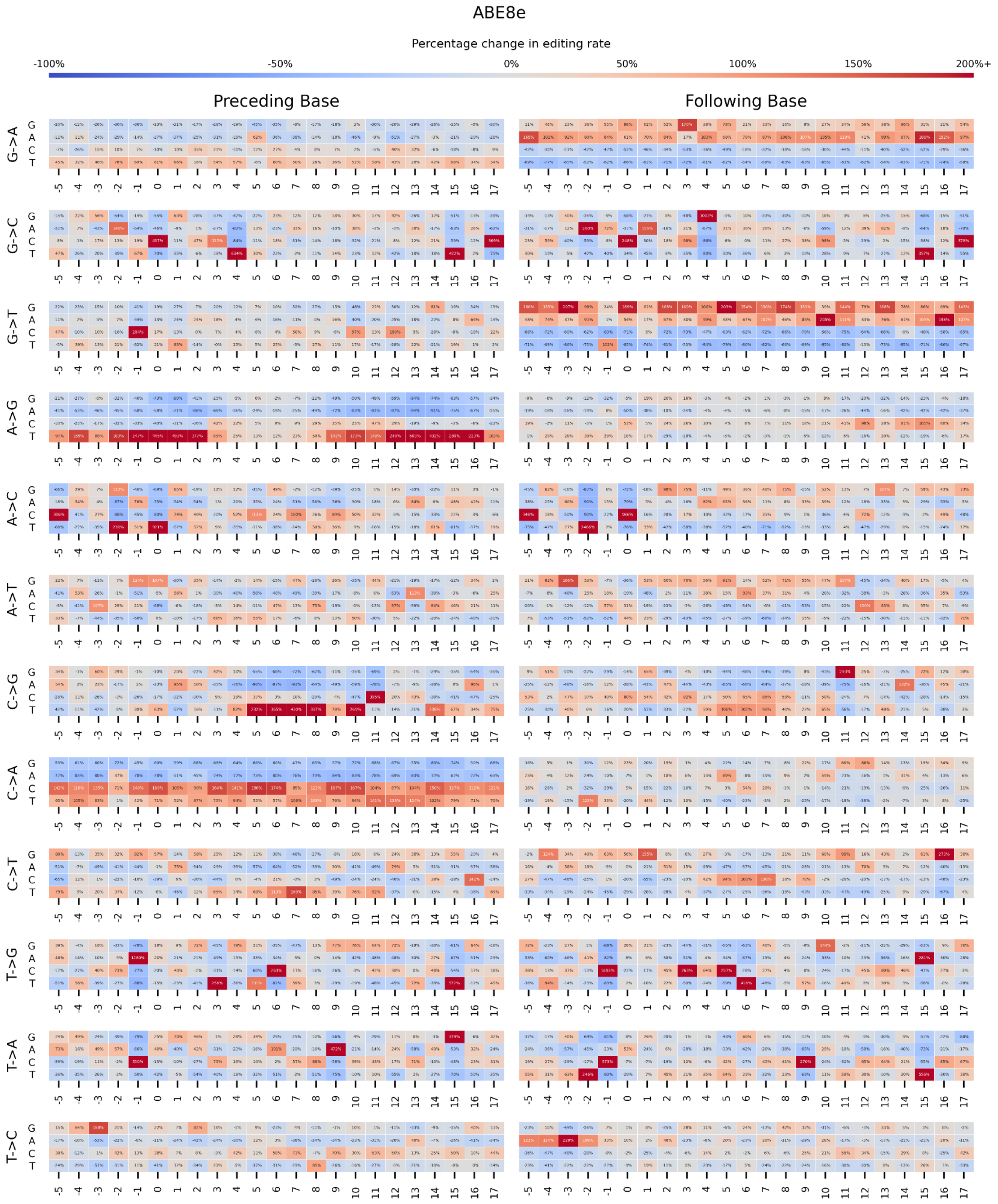

Position-dependent effect of the preceding and following base in ABE8e. Percentage change in editing rate (color) depending on the identity of the base (y-axis) preceding (left) or following (right) the target base in the sequence (x-axis) for each substitution type (rows).

Figure S2G

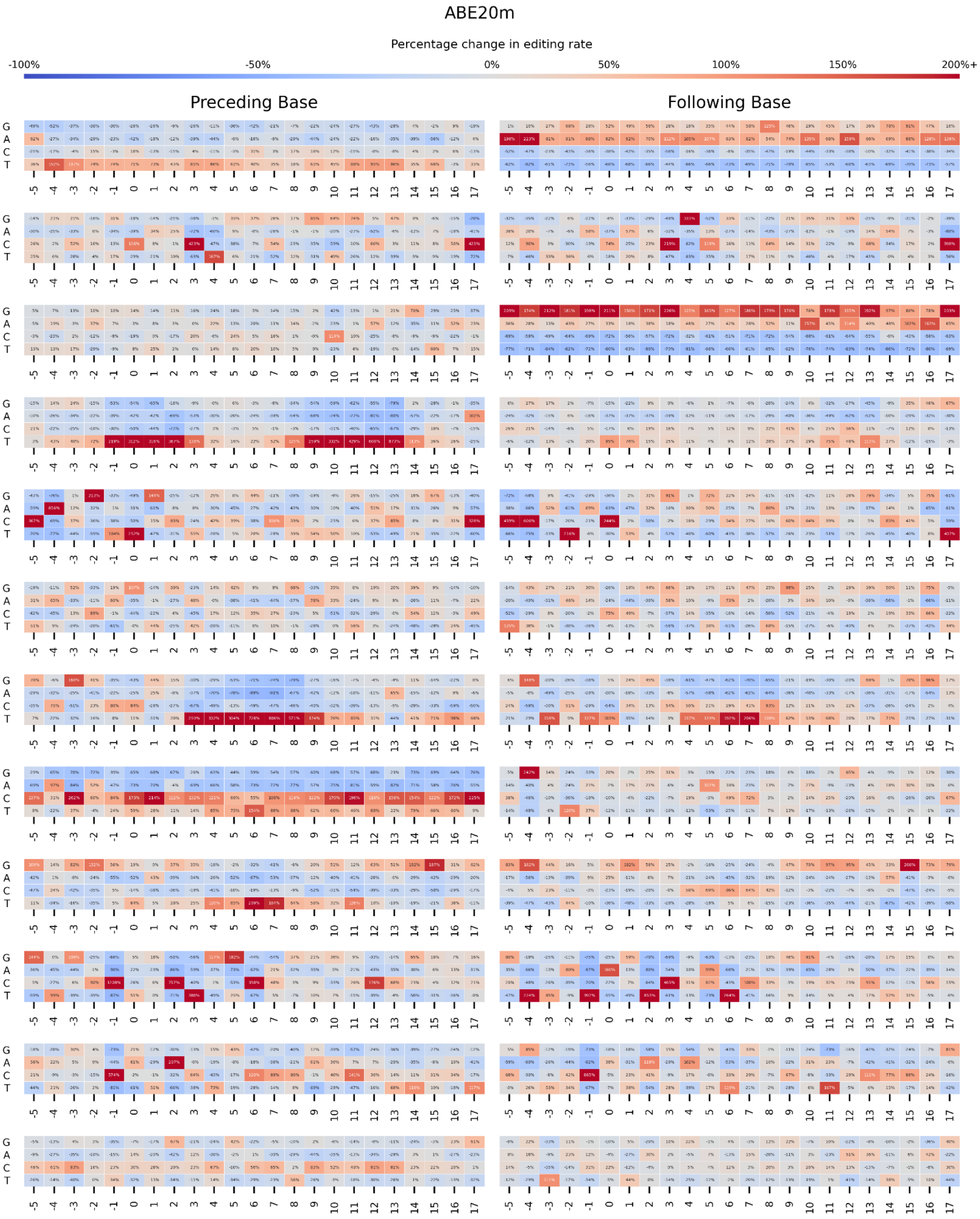

Position-dependent effect of the preceding and following base in ABE20m. Percentage change in editing rate (color) depending on the identity of the base (y-axis) preceding (left) or following (right) the target base in the sequence (x-axis) for each substitution type (rows).

Figure S3A

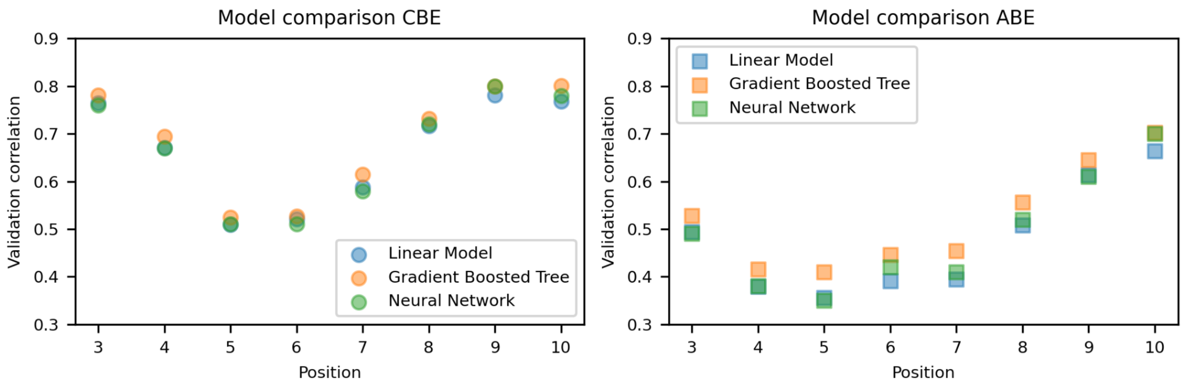

Comparison of model types on the positional prediction task. Average cross-validation correlation between measured and predicted values (y-axis) at each position in the guide (x-axis) for each type of model tested (colors).

Figure S3B

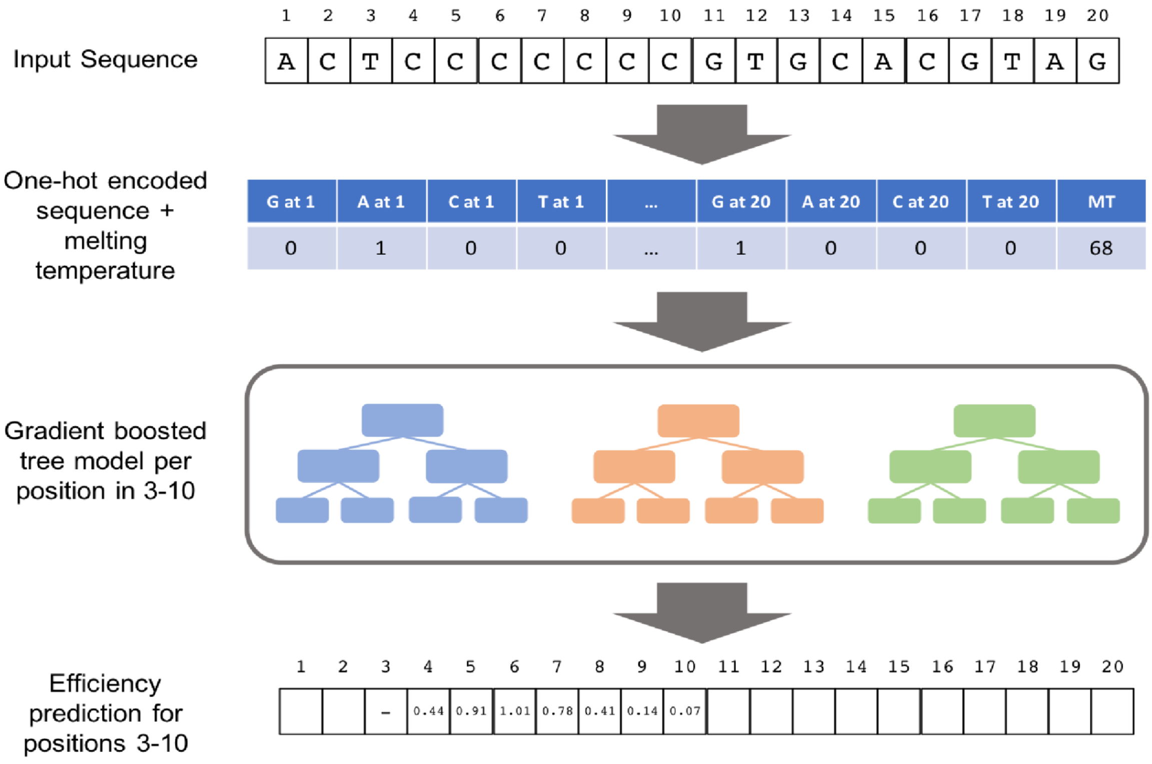

Outline of FORECasT-BE architecture. A 20 nucleotide input sequence is one-hot encoded into a feature vector and the melting temperature of the sequence is appended. A gradient boosted tree predictor uses these input features to predict a z-score representing editing efficiency for a given position in the target sequence. We train one gradient boosted tree predictor for each of positions 3-10.

Figure S3C

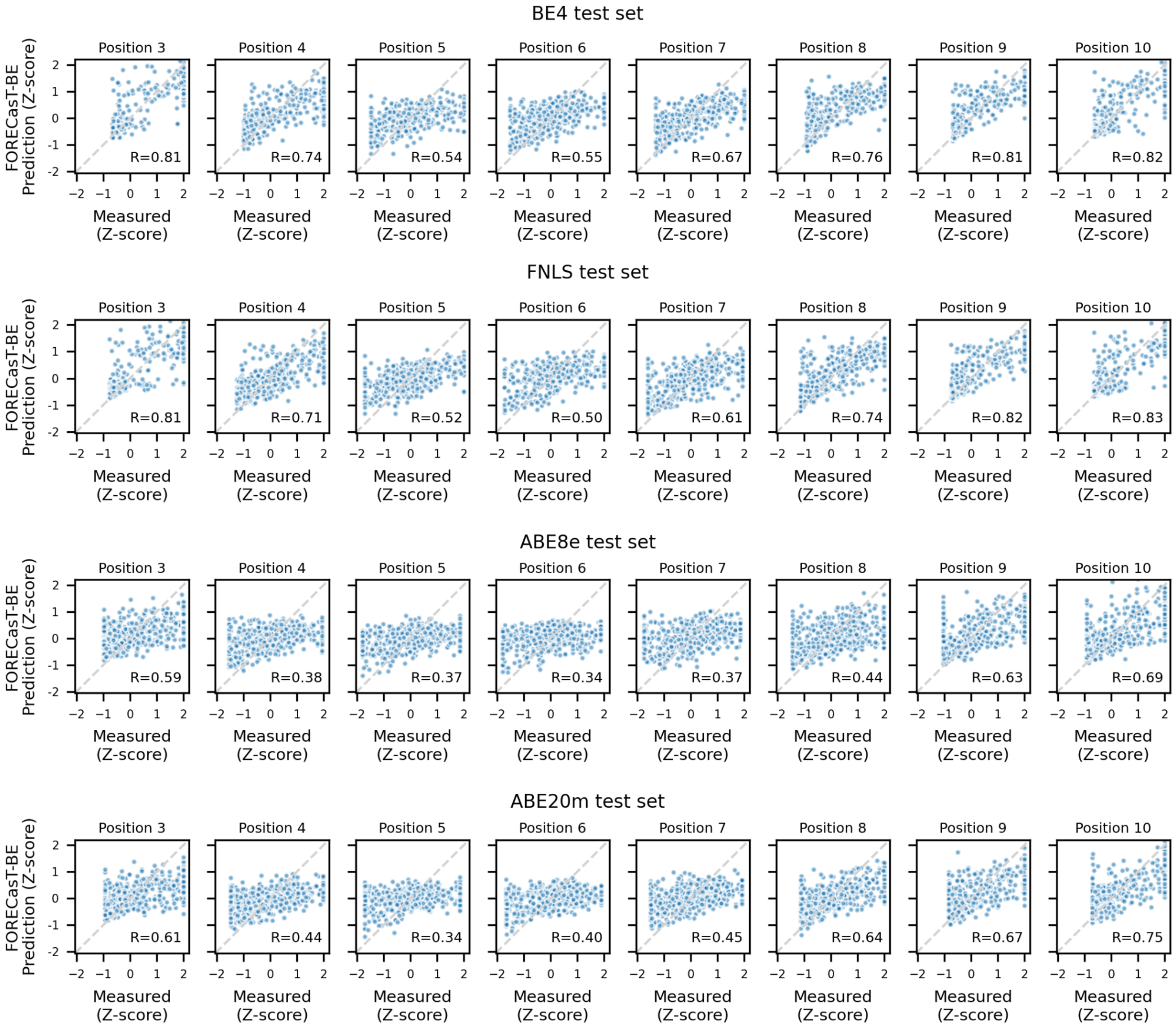

FORECasT-BE accurately predicts editing rate in multiple editors. Measured (x-axis) and predicted (y-axis) standardized editing rate (Methods) for guide RNAs (markers) with an adenine or cytosine at the different editing window positions (panels) for BE4 (top row), FNLS (second row), ABE8e (third row) and ABE20m (bottom row). Dashed line: y=x. Label: Pearson's R between measured and predicted scores.

Figure S3D

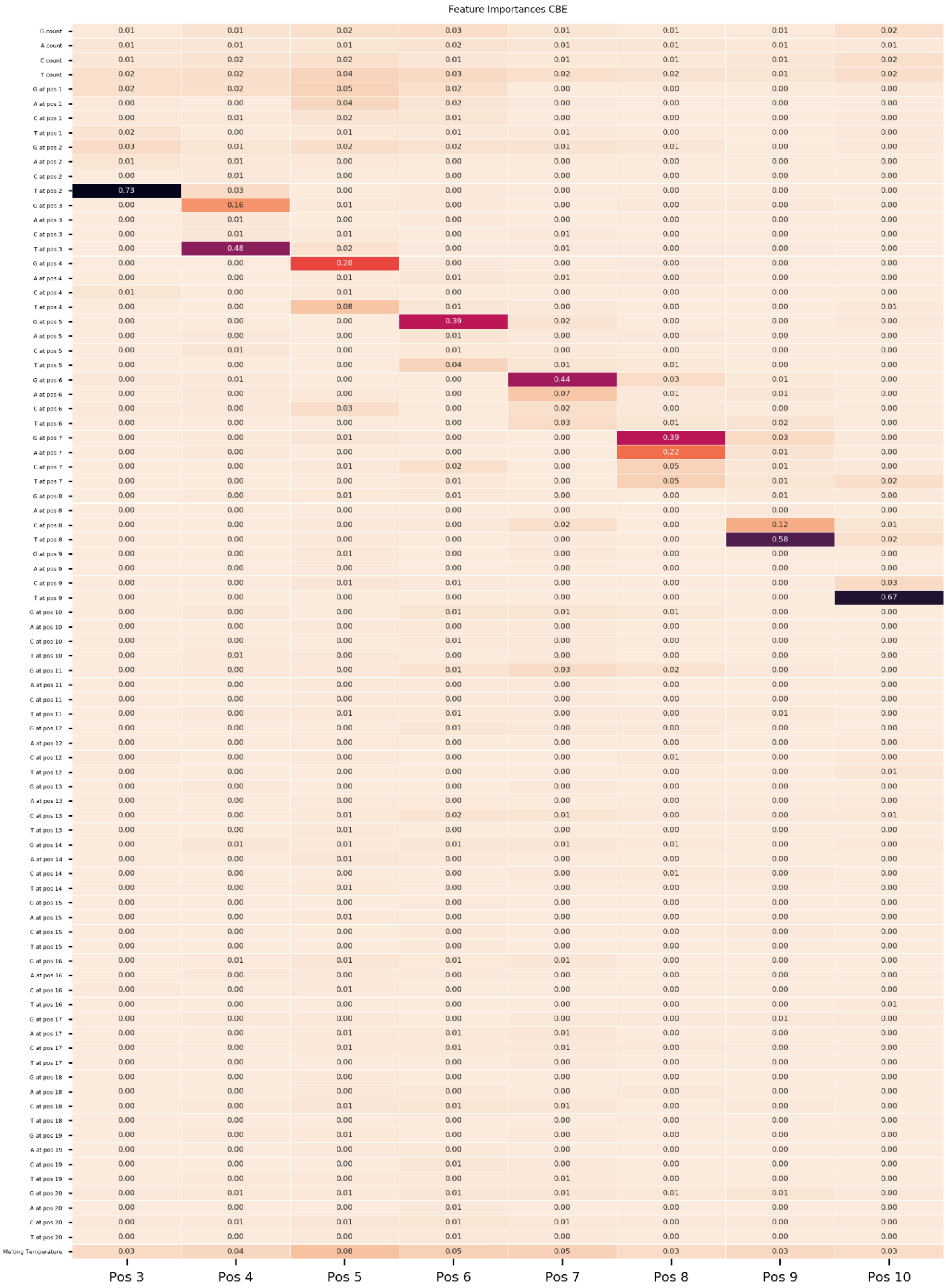

Feature importances for cytosine editors from FORECasT-BE. Gini coefficient (color) for every feature (y-axis) for each positional predictor (x-axis).

Figure S3E

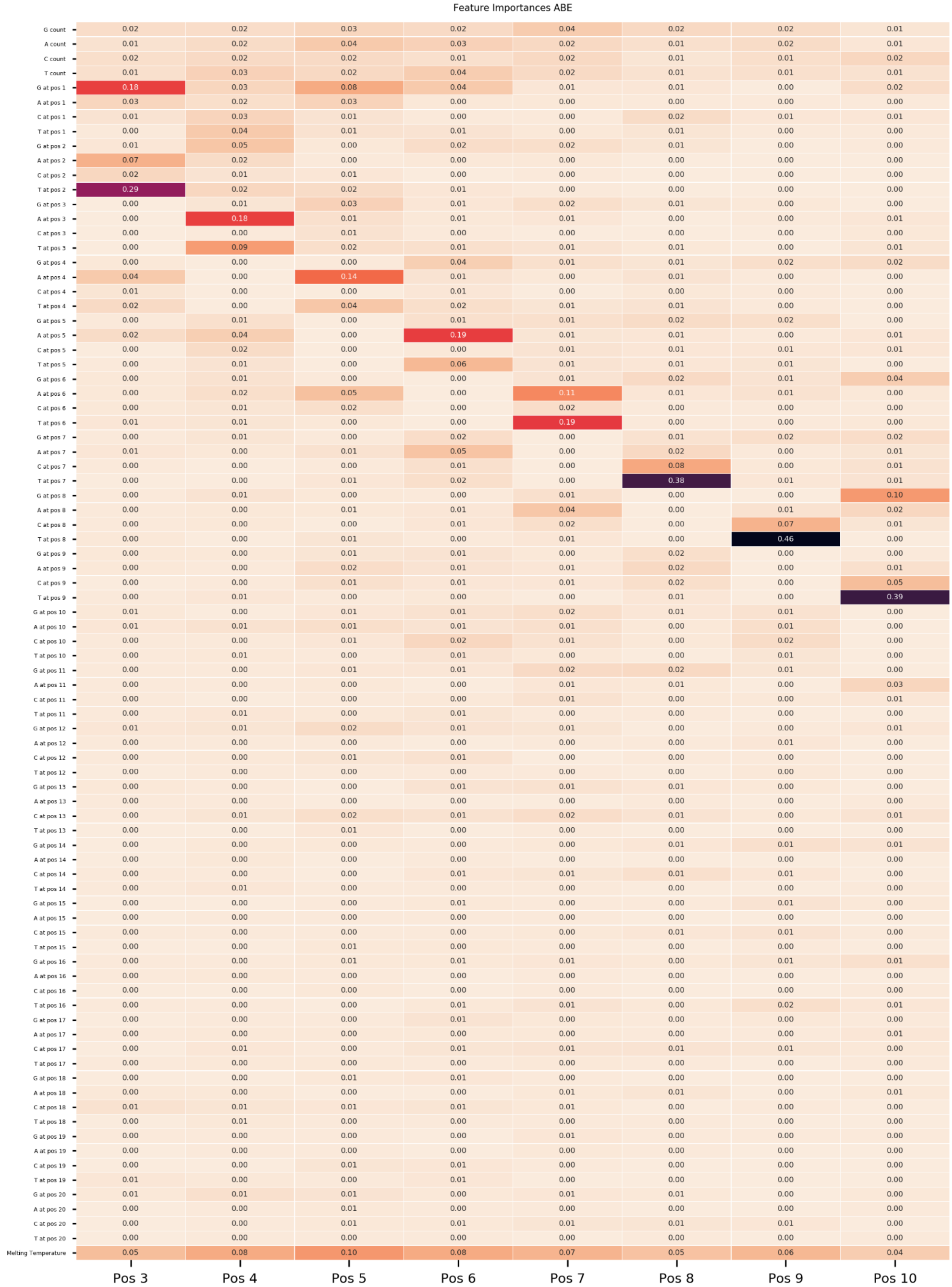

Feature importances for adenine editors from FORECasT-BE. Gini coefficient (color) for every feature (y-axis) for each positional predictor (x-axis).

Figure S3F

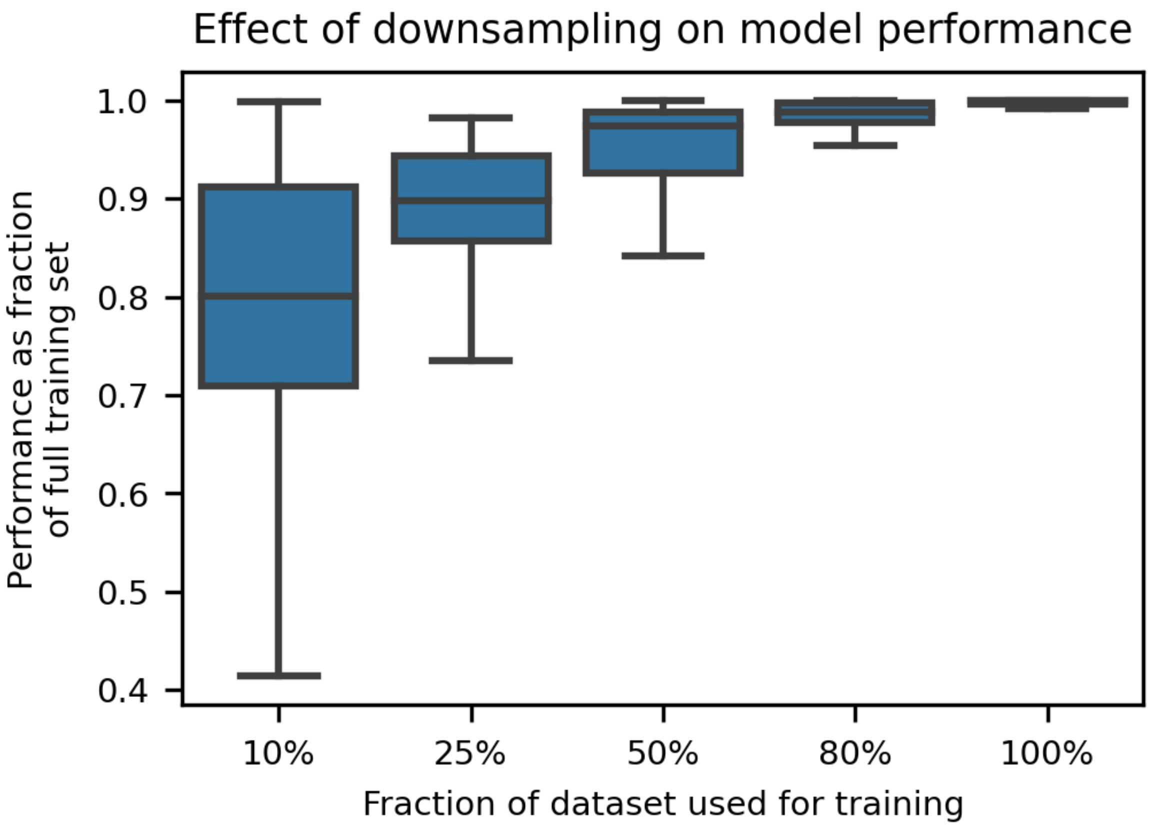

Downsampling experiments reveal that model performance plateaus when using at least 80% of the training data. Correlation between measured editing and predicted editing as a fraction of the correlation when using the full training set (y-axis) for models trained on 10%, 25%, 50% and 80% of training data (x-axis). Each model and downsampling threshold sampled 100 times. Data shown for models trained to predict editing at each position. Boxes: median and quartiles; whiskers: 1.5 interquartile ranges from the top and bottom quartiles (bounded by 0 and 1).

Figure S3G

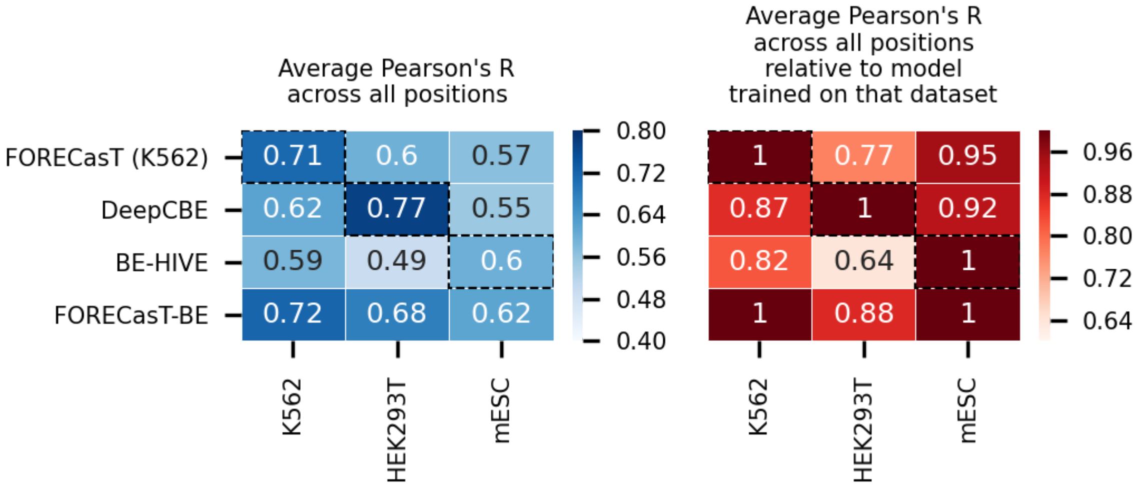

FORECasT-BE generalizes well to many datasets for cytosine editors. Average performance of models (y-axis) at all positions when evaluated on a test set from one cell type (x-axis), both raw (left, blue heatmap) and normalized relative to the performance of the model trained on that dataset (right, red heatmap). Models compared are: FORECasT model trained only on K562 cells (top row), DeepCBE trained on HEK293T cells (second row), BE-HIVE trained on mESC cells (third row) and FORECasT-BE model trained on multiple cell types (bottom row). Dotted outlines indicate that a model was trained using training data from that cell type.

Figure S3H

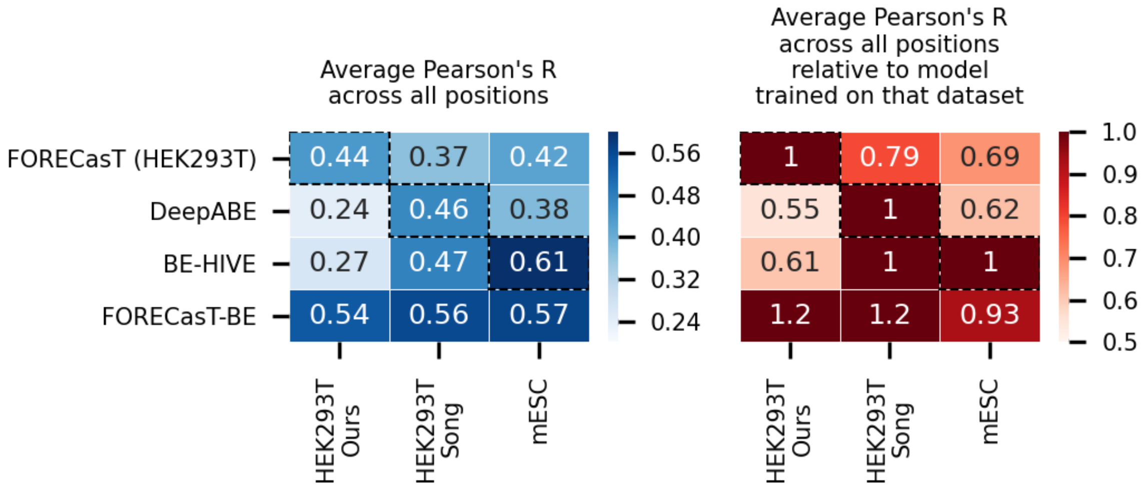

FORECasT-BE generalizes well to many datasets for adenine editors. Average performance of models (y-axis) at all positions when evaluated on a test set from one cell type (x-axis), both raw (left, blue heatmap) and normalized relative to the performance of the model trained on that dataset (right, red heatmap). Models compared are: FORECasT model trained only on HEK293T cells (top row), DeepABE trained on HEK293T cells (second row), BE-HIVE trained on mESC cells (third row) and FORECasT-BE model trained on multiple cell types (bottom row). Dotted outlines indicate that a model was trained using training data from that cell type.

Figure S3I

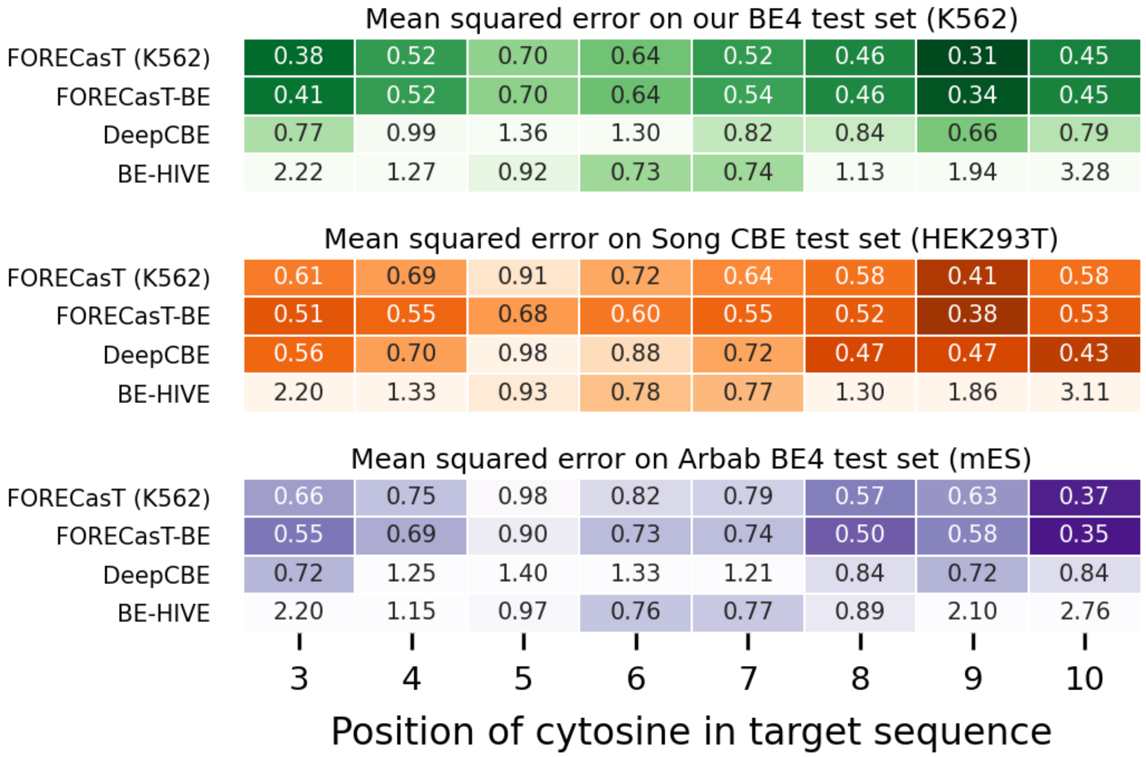

FORECasT-BE accurately predicts in a variety of contexts. Pearson's R between measurements and predictions for models trained on different cell types (y-axis) when using cytosine datasets with an editable cytosine at a given position in the guide sequence (x-axis). Models are evaluated on datasets from this study (K562, top heatmap), Song et al (HEK293T, middle) and Arbab et al (mESC, bottom). Models compared are: a FORECasT model trained on only K562 cells (top row), FORECasT-BE trained on multiple cell types (second row), DeepCBE trained on HEK293T cells (third row) and BE-HIVE trained on mES cells (bottom row).

Figure S3J

Mean squared error on our ABE8e test set (HEK293T)

	3	4	5	6	7	8	9	10
FORECasT (HEK293T)	0.65	0.86	0.96	0.86	0.89	0.84	0.62	0.51
FORECasT-BE	0.56	0.54	0.65	0.50	0.52	0.60	0.49	0.45
DeepABE	17.89	2.57	1.02	0.80	0.84	1.00	1.17	1.42
BE-HIVE	3.72	2.03	0.99	0.58	0.86	1.67	2.78	4.08

Mean squared error on Song ABE test set (HEK293T)

	3	4	5	6	7	8	9	10
FORECasT (HEK293T)	1.11	0.99	0.85	0.90	0.83	0.88	0.73	0.74
FORECasT-BE	0.85	0.81	0.73	0.69	0.72	0.69	0.63	0.59
DeepABE	16.07	2.14	0.77	0.69	0.92	0.90	0.98	1.36
BE-HIVE	3.27	1.58	0.69	0.75	0.73	1.31	2.53	3.73

Mean squared error on Arbab ABE test set (mES)

	3	4	5	6	7	8	9	10
FORECasT (HEK293T)	2.19	1.00	0.97	0.93	0.80	0.56	0.67	0.80
FORECasT-BE	1.76	0.90	0.82	0.74	0.67	0.46	0.54	0.66
DeepABE	13.78	2.98	1.16	1.05	1.04	0.82	0.63	1.06
BE-HIVE	4.66	1.89	0.82	0.71	0.75	1.10	2.51	3.92

Position of adenine in target sequence

FORECasT-BE accurately predicts in a variety of contexts. Pearson's R between measurements and predictions for models trained on different cell types (y-axis) when using adenine datasets with an editable adenine at a given position in the guide sequence (x-axis). Models are evaluated on datasets from this study (HEK293T, top heatmap), Song et al (HEK293T, middle) and Arbab et al (mESC, bottom). Models compared are: a FORECasT model trained on only HEK293T cells (top row), FORECasT-BE trained on multiple cell types (second row), DeepABE trained on HEK293T cells (third row) and BE-HIVE trained on mES cells (bottom row).

Figure S3K

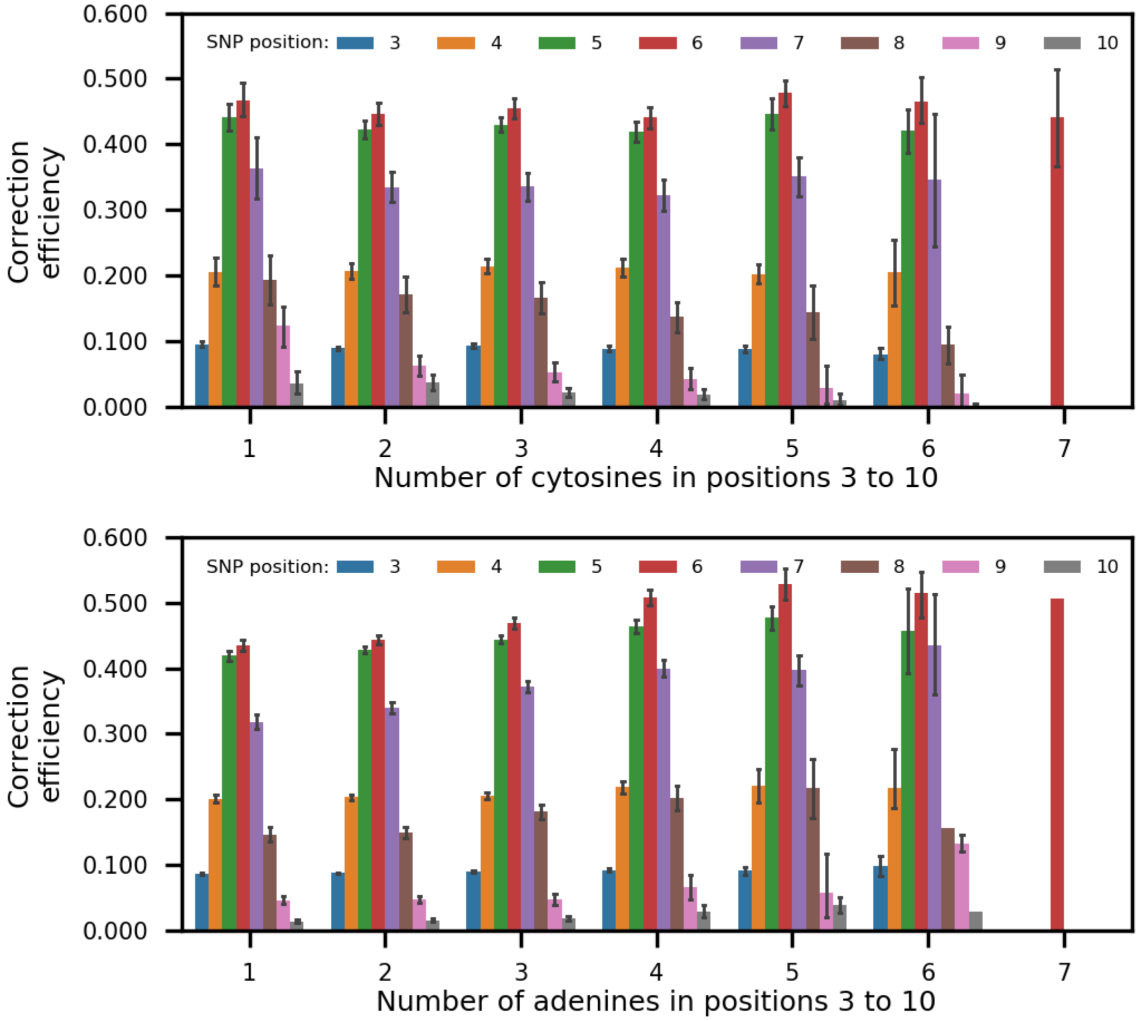

Correction efficiency varied across guides designed to correct pathogenic SNPs. Predicted efficiency to correct a pathogenic SNP (y-axis), for guides with differing numbers of cytosines (top) or adenines (bottom) in positions 3-10 (x-axis), and having SNPs at different positions in their sequence (colors). Error bars: 95% confidence intervals from 1000 bootstrap samples.

Figure S4A

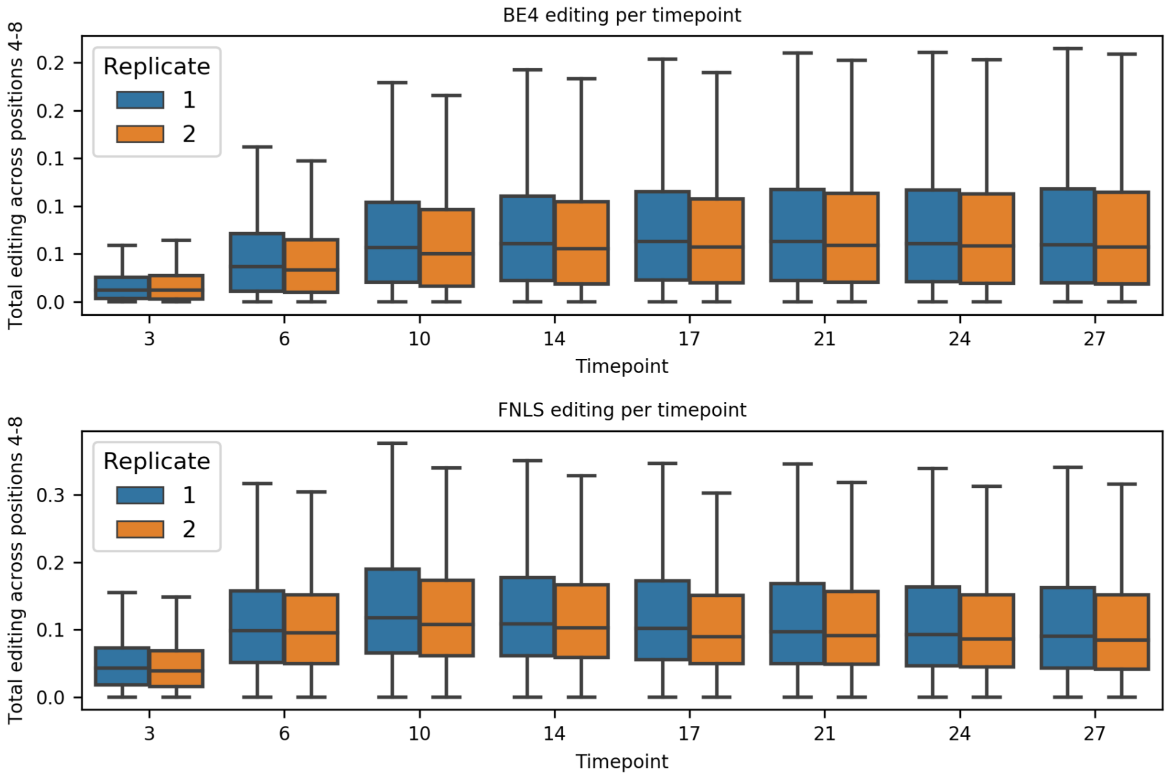

Editing plateaus at day 10, but slowly decreases afterward. Total editing measured across positions 4-8 (y-axis) for samples taken at each timepoint after infection in the screen (x-axis), for each replicate (colors). Boxes: median and quartiles; whiskers: 1.5 interquartile ranges from the top and bottom quartiles (bounded by 0 and 1)

Figure S4B

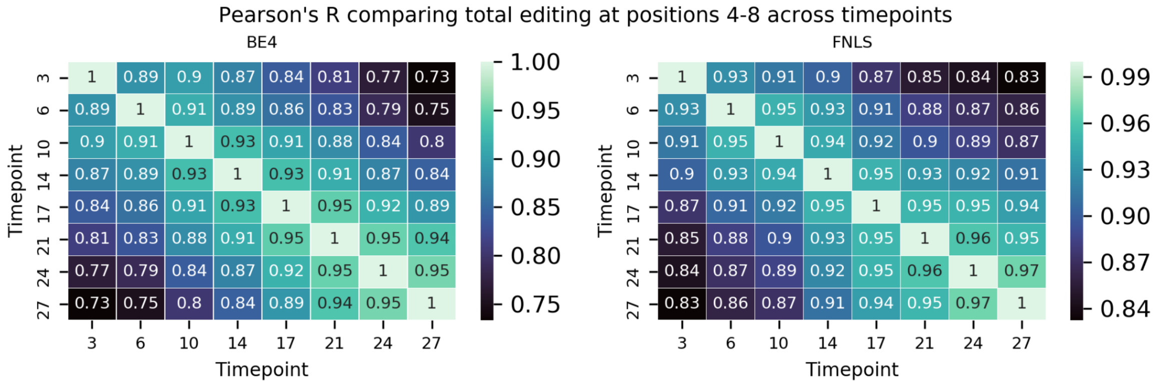

Closer timepoints edit similarly. Pearson's R between total editing at positions 4-8 at different timepoints (x- and y-axes) .

Figure S4C

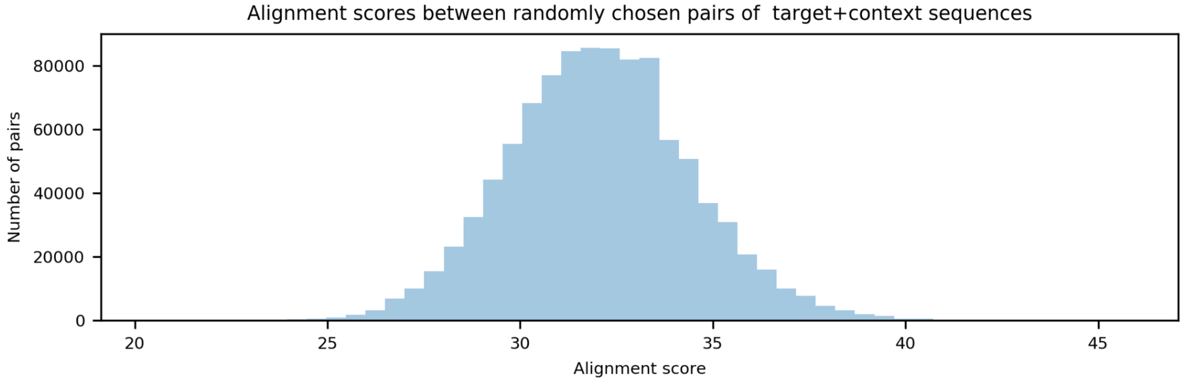

Alignment scores between randomly chosen 79nt target+context sequences in our constructs. Number of randomly chosen pairs of 79nt target+context sequences (y-axis) with a specific alignment score (x-axis).

Table S1

Primer	Sequence
P1	ACACTCTTTCCCTACACGACGCTCTTCCGATCTGACGTCCAGAGCACAGATGG
P2	TCGGCATTCCTGCTGAACCGCTCTTCCGATCTACCCGGTAGAATTGGATCCAAAC
P3	AATGATACGGCGACCACCGAGATCTACACTCTTTCCCTACACGACGCTCTTCCGATC*T
P4*	CAAGCAGAAGACGGCATACGAGATN$_{10}$GAGATCGGTCTCGGCATTCCTGCTGAACCGCTCTTCCGATC T
P5	ACACTCTTTCCCTACACGACGCTCTTCCGATCTCTTGGCTTTATATATCTTGTGGAAAGGACGAAACA
P6	TCGGCATTCCTGCTGAACCGCTCTTCCGATCTCTAAAGCGCATGCTCCAGACTGCC

*index for multiplexed sequencing

Primers sequences used as part of high throughput experiments

Table S2

Primer	Sequence
AARS1_2_gRNA_fw	CACCGGACTCTCAAATCCCTAAAGA
AARS1_2_gRNA_rv	AAACTCTTTAGGGATTTGAGAGTCC
AARS1_gRNA_fw	CACCGCACGCCCTGTGTTGTCAGGC
AARS1_gRNA_rv	AAACGCCTGACAACACAGGGCGTGC
DAD1_3_gRNA_fw	CACCGCTGACCGGGGCGCTGCAGTT
DAD1_3_gRNA_rv	AAACAACTGCAGCGCCCCGGTCAGC
DAD1_gRNA_fw	CACCGCGGTCAGCAGTATATACAGC
DAD1_gRNA_rv	AAACGCTGTATATACTGCTGACCGC
DDX3X_2_gRNA_fw	CACCGCAACCTCATTCTTTAACGAG

DDX3X_2_gRNA_rv	AAACCTCGTTAAAGAATGAGGTTGC
HARS1_1_gRNA_fw	CACCGGGCTCAGCATTGGGGTGGAG
HARS1_1_gRNA_rv	AAACCTCCACCCCAATGCTGAGCCC
HCFC1_10_gRNA_fw	CACCGAAGACCATGGCTGTGACACC
HCFC1_10_gRNA_rv	AAACGGTGTCACAGCCATGGTCTTC
HCFC1_14_gRNA_fw	CACCGCCAACTCCCTGGAGGTGAGC
HCFC1_14_gRNA_rv	AAACGCTCACCTCCAGGGAGTTGGC
HCFC1_16_gRNA_fw	CACCGCCTCCGTGTCCTCGACTCGG
HCFC1_16_gRNA_rv	AAACCCGAGTCGAGGACACGGAGGC
HCFC1_2_gRNA_fw	CACCGCCGTGGATCTGAGCAGCACA
HCFC1_2_gRNA_rv	AAACTGTGCTGCTCAGATCCACGGC
HCFC1_gRNA_fw	CACCGCCCCCTCCGTGTCCTCGACT
HCFC1_gRNA_rv	AAACAGTCGAGGACACGGAGGGGGC
KARS1_gRNA_fw	CACCGCTGCCCCCCACAGCTGGCTG
KARS1_gRNA_rv	AAACCAGCCAGCTGTGGGGGGCAGC
RCC1_2_gRNA_fw	CACCGGGAGCCCATGAAGAAGAGCA
RCC1_2_gRNA_rv	AAACTGCTCTTCTTCATGGGCTCCC
RCC1_3_gRNA_fw	CACCGCATTCCGGAGGATGTTGTGC
RCC1_3_gRNA_rv	AAACGCACAACATCCTCCGGAATGC
TAF1_3_gRNA_fw	CACCGGGCTGATGAAAACTTCCTGA
TAF1_3_gRNA_rv	AAACTCAGGAAGTTTTCATCAGCCC
hU6_fw	gagggcctatttcccatgattc
AARS1_2_fw	TTGTGGAAAGGACGAAACACCGCTGCAGTCATCCCCCAGTGG

AARS1_2_rv	ACTTGCTATTTCTAGCTCTAAAACACATCGGCTTTGCTGGCTCG
DAD1_3_fw	TTGTGGAAAGGACGAAACACCGGCGTCTGAAGTTGCTGGACG
DAD1_3_rv	ACTTGCTATTTCTAGCTCTAAAACAGAGTTGAAGGGGAAGGTCC
HCFC1_2_fw	TTGTGGAAAGGACGAAACACCGAGGTCCTGTCAGCCTCCCAG
HCFC1_2_rv	ACTTGCTATTTCTAGCTCTAAAACACCGCAGAGCCGGCAGACTC
HCFC1_10_fw	TTGTGGAAAGGACGAAACACCGACCCACGGTGCTGAGTGTCC
HCFC1_10_rv	ACTTGCTATTTCTAGCTCTAAAACGGAGGCCACCTTCACAGTGG
HCFC1_14_fw	TTGTGGAAAGGACGAAACACCGCCACCACCCCCAGCCCGAG
HCFC1_14_rv	ACTTGCTATTTCTAGCTCTAAAACGCTGGAGAAGGTAGCTGTCG
HCFC1_16_fw	TTGTGGAAAGGACGAAACACCGGGGAGTGGAAGAGACTCAAAG
HCFC1_16_rv	ACTTGCTATTTCTAGCTCTAAAACGTGGGCAACAAATGCTACCTG
DDX3X_2_fw	TTGTGGAAAGGACGAAACACCGATATACTTTTTTGGGAACTC
DDX3X_2_rv	ACTTGCTATTTCTAGCTCTAAAACAGCTTCAACAAGAAGATCCA
HARS1_1_fw	TTGTGGAAAGGACGAAACACCGGGCATGTTCGACCCCAAAGG
HARS1_1_rv	ACTTGCTATTTCTAGCTCTAAAACGACCTACCTCTAGTCTCTGT
TAF1_3_fw	TTGTGGAAAGGACGAAACACCGTTTAGGAAACTTGAGGAA
TAF1_3_rv	ACTTGCTATTTCTAGCTCTAAAACTCCCAGATGATATCATCCTC
RCC1_2_fw	TTGTGGAAAGGACGAAACACCGTCCCTCCCTTCTAGGACAAT
RCC1_2_rv	ACTTGCTATTTCTAGCTCTAAAACTTTACCACAGGCACATCCAG
RCC1_3_fw	TTGTGGAAAGGACGAAACACCGGTGAGAATGTGATGGAGAGG
RCC1_3_rv	ACTTGCTATTTCTAGCTCTAAAACTTGCTTAGACACACGGTGTG
AARS1_1_fw	TTGTGGAAAGGACGAAACACCGACGCTCGGACCATCACTGTG
AARS1_1_rv	ACTTGCTATTTCTAGCTCTAAAACGTGCCGCTCCAAACACCACC

HCFC1_1_fw	TTGTGGAAAGGACGAAACACCGGGAGTGGAAGAGACTCAAAG
HCFC1_1_rv	ACTTGCTATTTCTAGCTCTAAAACCAGGTAGCATTTGTTGCCCAC
DAD1_1_fw	TTGTGGAAAGGACGAAACACCGGCTCCACTCCGCAGCGTCTG
DAD1_1_rv	ACTTGCTATTTCTAGCTCTAAAACAAGGTCCCCACGAGGAGACAG
KARS_2_fw	TTGTGGAAAGGACGAAACACCGGCTCCACTCCGCAGCGTCTG
KARS_2_rv	ACTTGCTATTTCTAGCTCTAAAACAAGGTCCCCACGAGGAGACAG
UDI0001-F(i5)	AATGATACGGCGACCACCGAGATCTACACCTAGCGCTACACTCTTTCCCTACACGACGCTCTTCCGATCTTTGTGGAAAGGACGAAACACCG
UDI0003-F(i5)	AATGATACGGCGACCACCGAGATCTACACCGTCTGCGACACTCTTTCCCTACACGACGCTCTTCCGATCTGCTTGTGGAAAGGACGAAACACCG
UDI0004-F(i5)	AATGATACGGCGACCACCGAGATCTACACTACTCATAACACTCTTTCCCTACACGACGCTCTTCCGATCTAGCTTGTGGAAAGGACGAAACACCG
UDI0005-F(i5)	AATGATACGGCGACCACCGAGATCTACACACGCACCTACACTCTTTCCCTACACGACGCTCTTCCGATCTCAACTTGTGGAAAGGACGAAACACCG
UDI0006-F(i5)	AATGATACGGCGACCACCGAGATCTACACGTATGTTCACACTCTTTCCCTACACGACGCTCTTCCGATCTTGCACCTTGTGGAAAGGACGAAACACCG
UDI0019-F(i5)	AATGATACGGCGACCACCGAGATCTACACCATAGAGTACACTCTTTCCCTACACGACGCTCTTCCGATCTTTGTGGAAAGGACGAAACACCG
UDI0020-F(i5)	AATGATACGGCGACCACCGAGATCTACACTGCGAGACACACTCTTTCCCTACACGACGCTCTTCCGATCTCTTGTGGAAAGGACGAAACACCG
UDI0001-R(i7)	CAAGCAGAAGACGGCATACGAGATAACCGCGGGTGACTGGAGTTCAGACGTGTGCTCTTCCGATCTACTTGCTATTTCTAGCTCTAAAAC
UDI0003-R(i7)	CAAGCAGAAGACGGCATACGAGATCCAAGTCCGTGACTGGAGTTCAGACGTGTGCTCTTCCGATCTACTTGCTATTTCTAGCTCTAAAAC
UDI0004-R(i7)	CAAGCAGAAGACGGCATACGAGATTTGGACTTGTGACTGGAGTTCAGACGTGTGCTCTTCCGATCTACTTGCTATTTCTAGCTCTAAAAC
UDI0005-R(i7)	CAAGCAGAAGACGGCATACGAGATCAGTGGATGTGACTGGAGTTCAGACGTGTGCTCTTCCGATCTACTTGCTATTTCTAGCTCTAAAAC
UDI0006-R(i7)	CAAGCAGAAGACGGCATACGAGATTGACAAGCGTGACTGGAGTTCAGACGTGTGCTCTTCCGATCTACTTGCTATTTCTAGCTCTAAAAC
UDI0019-R(i7)	CAAGCAGAAGACGGCATACGAGATACTTACATGTGACTGGAGTTCAGACGTGTGCTCTTCC

	GATCTACTTGCTATTTCTAGCTCTAAAAC
UDI0020-R(i7)	CAAGCAGAAGACGGCATACGAGATGTCCGTGCGTGACTGGAGTTCAGACGTGTGCTCTTCCGATCTACTTGCTATTTCTAGCTCTAAAAC

Primers sequences used to amplify target regions in Target-AID screens.

Table S3

Position	Scaling factor for mean	Scaling factor for standard deviation
3	0.197	0.172
4	0.444	0.521
5	0.912	0.774
6	1.0	1.0
7	0.785	1.051
8	0.414	1.022
9	0.144	0.804
10	0.071	0.369

Scaling factors for means and standard deviations at each position. These are used to scale standardized predictions at each position appropriately when given only a single mean and a single standard deviation for an experiment.

Table S4

SANGER SAMPLE ID	SAMPLE LABEL	ERS	SAMPLE DESCRIPTION
T227_CRISPR8015850	22_1_3D14	ERS3536251	ABERA Replicate 1 Day 14
T227_CRISPR8015851	22_1_3D17A	ERS3536252	ABERA Replicate 1 Day 17A
T227_CRISPR8015852	22_1_3D17B	ERS3536253	ABERA Replicate 1 Day 17B
T227_CRISPR8015853	22_1_3D17C	ERS3536254	ABERA Replicate 1 Day 17C
T227_CRISPR8015854	22_1_3D17D	ERS3536255	ABERA Replicate 1 Day 17D
T227_CRISPR8015855	22_1_3D21	ERS3536256	ABERA Replicate 1 Day 21

T227_CRISPR8015856	22_1_3D24	ERS3536257	ABERA Replicate 1 Day 24
T227_CRISPR8015857	22_1_3D27	ERS3536258	ABERA Replicate 1 Day 27
T227_CRISPR8015858	22_1_4D14	ERS3536259	ABERA Replicate 2 Day 14
T227_CRISPR8015859	22_1_4D21	ERS3536260	ABERA Replicate 2 Day 21
T227_CRISPR8015860	22_1_4D24	ERS3536261	ABERA Replicate 2 Day 24
T227_CRISPR8015861	22_1_4D27	ERS3536262	ABERA Replicate 2 Day 27
T227_CRISPR8015862	22_1_5D3	ERS3536263	BE4 Replicate 1 Day 3
T227_CRISPR8015863	22_1_5D6	ERS3536264	BE4 Replicate 1 Day 6
T227_CRISPR8015864	22_1_5D10	ERS3536265	BE4 Replicate 1 Day 10
T227_CRISPR8015865	22_1_5D14	ERS3536266	BE4 Replicate 1 Day 14
T227_CRISPR8015866	22_1_5D17	ERS3536267	BE4 Replicate 1 Day 17
T227_CRISPR8015867	22_1_5D21	ERS3536268	BE4 Replicate 1 Day 21
T227_CRISPR8015868	22_1_5D24	ERS3536269	BE4 Replicate 1 Day 24
T227_CRISPR8015869	22_1_5D27	ERS3536270	BE4 Replicate 1 Day 27
T227_CRISPR8015870	22_1_6D3	ERS3536271	BE4 Replicate 2 Day 3
T227_CRISPR8015871	22_1_6D6	ERS3536272	BE4 Replicate 2 Day 6
T227_CRISPR8015872	22_1_6D10	ERS3536273	BE4 Replicate 2 Day 10
T227_CRISPR8015873	22_1_6D14	ERS3536274	BE4 Replicate 2 Day 14
T227_CRISPR8015874	22_1_6D17	ERS3536275	BE4 Replicate 2 Day 17
T227_CRISPR8015875	22_1_6D21	ERS3536276	BE4 Replicate 2 Day 21
T227_CRISPR8015876	22_1_6D24	ERS3536277	BE4 Replicate 2 Day 24
T227_CRISPR8015877	22_1_6D27	ERS3536278	BE4 Replicate 2 Day 27
T227_CRISPR8015878	22_1_7D3	ERS3536279	FNLS Replicate 1 Day 3
T227_CRISPR8015879	22_1_7D6	ERS3536280	FNLS Replicate 1 Day 6
T227_CRISPR8015880	22_1_7D10	ERS3536281	FNLS Replicate 1 Day 10
T227_CRISPR8015881	22_1_7D14	ERS3536282	FNLS Replicate 1 Day 14

T227_CRISPR8015882	22_1_7D17	ERS3536283	FNLS Replicate 1 Day 17
T227_CRISPR8015883	22_1_7D21	ERS3536284	FNLS Replicate 1 Day 21
T227_CRISPR8015884	22_1_7D24	ERS3536285	FNLS Replicate 1 Day 24
T227_CRISPR8015885	22_1_7D27	ERS3536286	FNLS Replicate 1 Day 27
T227_CRISPR8015886	22_1_8D3	ERS3536287	FNLS Replicate 2 Day 3
T227_CRISPR8015887	22_1_8D6	ERS3536288	FNLS Replicate 2 Day 6
T227_CRISPR8015888	22_1_8D10	ERS3536289	FNLS Replicate 2 Day 10
T227_CRISPR8015889	22_1_8D14	ERS3536290	FNLS Replicate 2 Day 14
T227_CRISPR8015890	22_1_8D17	ERS3536291	FNLS Replicate 2 Day 17
T227_CRISPR8015891	22_1_8D21	ERS3536292	FNLS Replicate 2 Day 21
T227_CRISPR8015892	22_1_8D24	ERS3536293	FNLS Replicate 2 Day 24
T227_CRISPR8015893	22_1_8D27	ERS3536294	FNLS Replicate 2 Day 27

Accession numbers for sequencing data from this experiment. These are all under ENA project PRJEB12405.

Table S5

(provided as a supplementary file)

Sequences of 108 cytosine-targeting guides and 421 adenine-targeting guides that were predicted to correct disease-relevant mutations from ClinVar with high efficiency and over 80% purity.

Table S6

(provided as a supplementary file)

Positional edit rates at 15 target sites screened using the TARGET-AID editor.
